# Automated affinity selection for rapid discovery of peptide binders†

**DOI:** 10.1039/d1sc02587b

**Published:** 2021-07-14

**Authors:** Genwei Zhang, Chengxi Li, Anthony J. Quartararo, Andrei Loas, Bradley L. Pentelute

**Affiliations:** Department of Chemistry, Massachusetts Institute of Technology 77 Massachusetts Avenue Cambridge MA 02139 USA blp@mit.edu; The Koch Institute for Integrative Cancer Research, Massachusetts Institute of Technology 500 Main Street Cambridge MA 02142 USA; Center for Environmental Health Sciences, Massachusetts Institute of Technology 77 Massachusetts Avenue Cambridge MA 02139 USA; Broad Institute of MIT and Harvard 415 Main Street Cambridge MA 02142 USA

## Abstract

In-solution affinity selection (AS) of large synthetic peptide libraries affords identification of binders to protein targets through access to an expanded chemical space. Standard affinity selection methods, however, can be time-consuming, low-throughput, or provide hits that display low selectivity to the target. Here we report an automated bio-layer interferometry (BLI)-assisted affinity selection platform. When coupled with tandem mass spectrometry (MS), this method enables both rapid *de novo* discovery and affinity maturation of known peptide binders with high selectivity. The BLI-assisted AS-MS technology also features real-time monitoring of the peptide binding during the library selection process, a feature unattainable by current selection approaches. We show the utility of the BLI AS-MS platform toward rapid identification of novel nanomolar (dissociation constant, *K*_D_ < 50 nM) non-canonical binders to the leukemia-associated oncogenic protein menin. To our knowledge, this is the first application of BLI to the affinity selection of synthetic peptide libraries. We believe our approach can significantly accelerate the use of synthetic peptidomimetic libraries in drug discovery.

## Abstract 2

This work reports an automated affinity selection-mass spectrometry (AS-MS) approach amenable to both *de novo* peptide binder discovery and affinity maturation of known binders in a high-throughput and selective manner.

## Graphical abstract

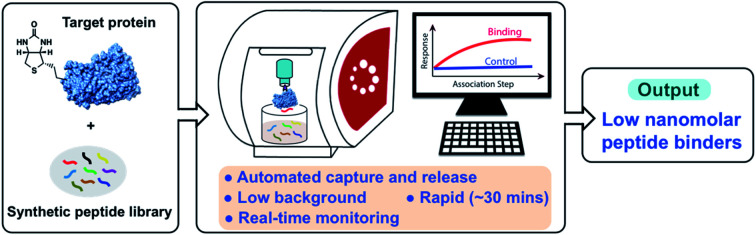

## Introduction

Protein–protein interactions (PPIs) are critical for cellular signal transduction, metabolism regulation, and cell cycles.^1^ Molecules that disrupt PPIs are important precursors for, among others, cancer therapeutics. Over the past two decades, peptide-based PPI inhibitors continued to attract interest.^2–11^ A variety of techniques were developed for peptide binder discovery, such as high-throughput screening (HTS),^12,13^ fragment-based^14^ and structure-based^15–20^ design.

When compared to small-molecule drugs (<500 Da) and large biologics (>150 000 Da), the advantages of peptide-based therapeutics reside not only in good efficacy, safety and tolerability, but also low immunogenicity and high selectivity.^21,22^ Moreover, the chemically diverse and relatively flexible peptide scaffolds can enhance the engagement of extended protein interface segments containing multiple “hot spots”, making them suitable for disruption of PPIs. Importantly, the incorporation of non-canonical amino acids allows peptide mimetics with increased structural diversity, yielding variants with high binding affinity and improved metabolic stability, thus potentially accelerating research and development of peptide pharmaceutics.^23,24^ Thus far, the US Food and Drug Administration (FDA) has approved over 60 peptide drugs and there are more than 150 peptides currently being investigated in clinical trials.^25^

Target-focused discovery tools play a critical role in the development of peptide-based binders. Traditional affinity selection (AS) approaches such as phage display^26^ and mRNA display^27^ can efficiently sample large library diversities. However, laborious preparation and the lack of high-level chemical control over the synthesis and selection processes are potential shortcomings of these genetically encoded approaches. Screening on-bead chemically synthesized libraries such as one-bead-one-compound (OBOC)^28^ can alleviate these drawbacks.^29,30^ Broader application of the OBOC method remains limited by relatively small library diversities and high nonspecific binding signals.^31^

To meet the increasing demand for high-throughput peptide drug discovery, we attempted to streamline the laborious experimentation during the affinity selection step by automating the workflow. Inspired by the concept of bio-layer interferometry (BLI),^32,33^ which is typically implemented on a programmable Octet instrument to characterize biomolecule interactions,^34^ we hypothesized that the BLI biosensor can serve as a solid capture support to realize an efficient, automated “peptide hit generator” when coupled with high-resolution nanoscale liquid chromatography-tandem mass spectrometry (nLC-MS/MS).

Here, after integrating the BLI advantages, *i.e.*, programmable, versatile, and label-free, we developed an automated affinity selection platform for rapid screening and optimization of peptide binders. We show that BLI-assisted AS-MS enables rapid *de novo* binder discovery to targeted proteins from synthetic randomized libraries with a diversity of up to 10^7^ members. Using BLI-assisted AS-MS, we developed a series of peptidomimetics targeting the MLL-rearranged leukemia oncogenic protein menin. The binding affinity of the optimized hit was improved by ∼200-fold in comparison with the parent canonical binding motif after incorporation of non-canonical amino acids. Our approach provides automated binder capture and release, low background with high reproducibility and real-time monitoring. We believe our method represents an important advancement to the existing combinatorial toolbox for the discovery of peptide-based therapeutics.

## Results

**Fig. 1:**
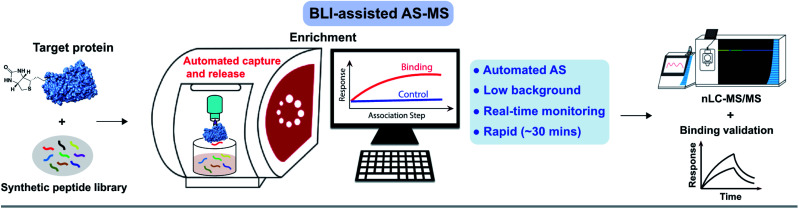
Affinity selection-mass spectrometry (AS-MS) assisted by bio-layer interferometry enables rapid screening of peptide combinatorial libraries. Schematic of the BLI-assisted AS-MS platform developed in this work. The target protein was biotinylated and loaded onto a streptavidin (SA)-coated biosensor tip and then sampled in synthetic peptide libraries to capture peptide binders with real-time monitoring of the association signal. Subsequently, bound peptides were eluted and then sequenced by nLC-MS/MS. Individual hits were synthesized and validated at the final step. Detailed experimental procedures of the BLI-assisted AS-MS can be found in the ESI.†

To efficiently and rapidly discover *de novo* peptide binders, we developed a novel affinity selection platform that combines the advantages of BLI and nLC-MS/MS for peptide sequencing (Fig. 1). This platform offers automated library screening capabilities with real-time monitoring of the protein loading and peptide binding steps on instruments utilizing the Octet assay,^3,34^ while significantly improving AS-MS selectivity through target-specific enrichment on the biosensor.

Our goal was to establish a rapid affinity selection platform that can reduce the high background signals, such as when using the traditional one-bead-one-compound screening^31^ and in the meantime, automates the workflow to reduce bench work time. Bio-layer interferometry is a label-free technology measuring biomolecular interactions with an optimized biosensor tip for ligand immobilization. We utilized commercially available streptavidin-coated biosensors to differentiate protein-bound *versus* unbound peptides. Bound peptides were next eluted and sequenced by nLC-MS/MS. Individual hits were then synthesized and the binding was validated.

### Streptavidin-coated BLI biosensor recovers 12ca5 peptide binders with high selectivity

To demonstrate the feasibility of using streptavidin (SA)-coated biosensors towards enhancing recovery of protein-specific peptide binders to sufficient amounts (>1.0 pg) for nLC-MS/MS sequencing, the anti-hemagglutinin (HA) monoclonal antibody 12ca5 and its binding peptide HA tag (amino acid sequence: YPYDVPDYAK) were chosen for experimental analysis. An additional lysine was installed at the peptide C-terminus to facilitate sequence fragmentation and improve sequence decoding confidence.^35^

**Fig. 2:**
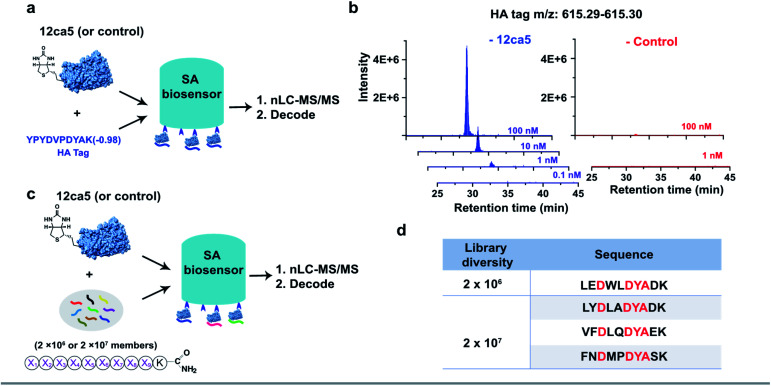
BLI-assisted AS-MS recovers high-affinity binders from both simple mixtures and randomized libraries. The schematic representation of (a) single HA tag recovery and (c) peptide hit discovery from randomized libraries against the 12ca5 protein using BLI-assisted AS-MS; experimental details can be found in the ESI.† (b) Extracted ion chromatogram (EIC) of recovered HA tag with a parallel selection against a control protein, human ACE2. (d) The list of HA binding motif-containing peptides screened from randomized libraries against 12ca5 protein using BLI-assisted AS-MS. The HA binding motif, D**DYA, was highlighted in red, and these peptides were not found in the control screening against ACE2 protein.

We first performed a ‘direct capture’ experiment. Briefly, the biotinylated 12ca5 protein was immobilized on the SA biosensor tip before dipping into the HA tag solutions prepared at the following concentrations: 100, 10, 1.0 and 0.1 nM (Fig. 2a). The 12ca5-bound peptides were then eluted with 6.0 M guanidine hydrochloride for further analysis. After nLC-MS/MS and sequence decoding, the expected ion (*m*/*z*: 615.29–615.30) was found enriched in the 100, 10 and 1.0 nM selection conditions. The ion intensities increased with concentration, indicating a dose-dependent ion enrichment (Fig. 2b). However, no detectable ion enrichment was found at 100 pM concentration, indicating a sensitivity cut-off point in terms of mass spectrometry sequencing (Fig. 2b).

In an ‘indirect capture’ experiment, the biotinylated 12ca5 protein was pre-mixed in solution with the HA tag prior to capture by the SA biosensor. Similarly, we found the detection threshold of the indirect capture approach to be 1.0 nM (Fig. S3†). Of note, the HA tag was not captured either by the ACE2 control protein or the biosensor alone even at the highest tested concentration (1000 nM from Fig. S3†), illustrating high target selectivity. Taken together, these results demonstrated that one single biosensor tip could provide sufficient ion enrichment for nLC-MS/MS sequencing.

### BLI-assisted AS-MS enriches 12ca5-specific binders from both 10^6^- and 10^7^-member randomized libraries

We assessed whether the BLI-assisted AS-MS approach can recover peptide binders from a complex mixture and enrich sufficient material for downstream nLC-MS/MS sequencing. We synthesized three peptide libraries comprised of 2 × 10^6^, 2 × 10^7^ and 2 × 10^8^ members, respectively, all with the design of (X)_9_K using split-and-pool peptide synthesis (Fig. 2c). X represents 18 natural amino acids except Cys because it could form intra- or inter-molecular disulfides and Ile because it is isobaric in mass with Leu. Selection against 12ca5 was then performed using these three synthetic peptide libraries, in which the per-member concentrations, due to overall peptide library solubility, decreased as the library diversity increased. The estimated theoretical concentrations per library peptide used in our study are 1.0 nM (2 × 10^6^-membered library), 100 pM (2 × 10^7^-membered library) and 10 pM (2 × 10^8^-membered library).

From the 2 × 10^6^ library, only one peptide (sequence: LEDWLDYAK) that matched the library design was identified (Fig. 2d). This peptide contains the HA binding motif (D**DYA), indicating a 12ca5-specific binder. When the library diversity increased 10-fold to 2 × 10^7^ members and the per-member concentration decreased 10-fold, three peptides that matched the library design were identified, and all three peptide hits contained the HA binding motif (Fig. 2d). Importantly, beyond these three peptides, all other decoded peptide sequences were found to not match the library design. Interestingly, we did not find any peptide hits that matched the library design from the 2 × 10^8^ library. Taken together, BLI-assisted AS-MS enables rapid discovery of high-affinity peptide binders from randomized libraries with high selectivity down to a concentration per library member of approximately 100 pM.

### Optimization of a menin binding motif sequence improves the binding affinity by 200-fold

The N-terminal MLL (mixed-lineage leukemia) protein fragment (sequence: SARWRFPARPGT) was previously reported essential for binding its oncogenic cofactor, menin.^36^ Multiple lines of evidence also demonstrated the middle residues (either RWRFP^36^ or FPARP^37^) are essential for the menin–MLL interaction. To further improve the binding affinity of peptide disruptors of the menin–MLL PPI, we leveraged our platform for rapid optimization of the menin binding motif 1 (MBM1, sequence: RWRFPARP) with unnatural (non-canonical) amino acids. To facilitate the subsequent ion fragmentation and sequence identification, an additional positively charged residue, l-arginine, was installed at the C-terminus.

**Fig. 3:**
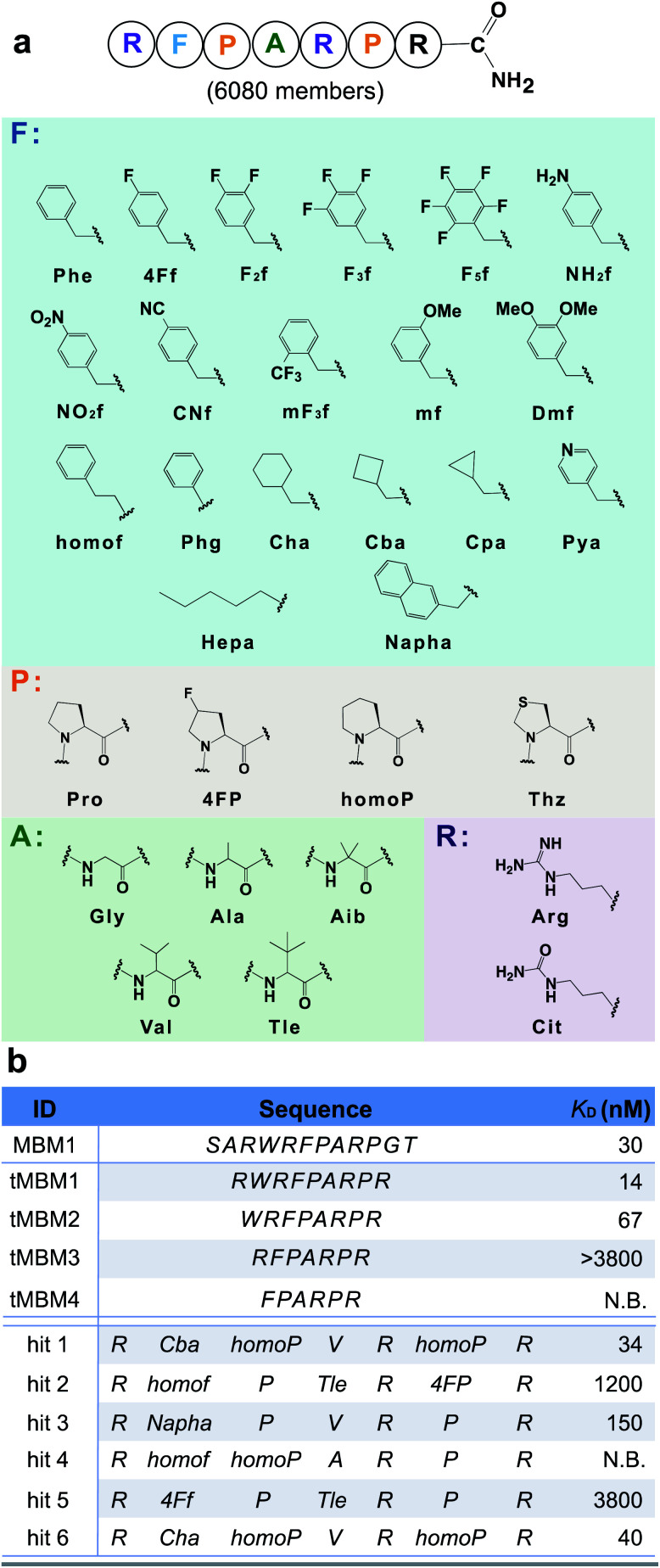
Unnatural amino acids improve the binding affinity of a menin-binding motif by 100-fold. (a) Library design based on the tMBM3 sequence. Phe-like monomers (cyan box) were installed into the F spot, and similarly, a list of monomers was chosen for the P spot (orange box), the A spot (green box), and R spot (purple box). An additional arginine was incorporated at the C-terminus to facilitate ion fragmentation and decoding. (b) Sequences and experimentally determined binding affinities for both the menin binding motif truncations and library hits, see Fig. S16† for experimental details. N.B. indicates no detectable binding.

To potentiate the potential advantages of short (<10-*mer*) peptides in terms of bioavailability and cellular penetration, we performed N-terminal truncations of the MBM1 sequence. A minimal length 7-*mer* binder (tMBM3) was discovered which retains menin-binding ability (apparent dissociation constant, *K*_D_ > 3.8 μM) and was chosen as the candidate for further optimization (Fig. 3).

We utilized tMBM3 as the parent sequence to design and synthesize a focused library for affinity maturation (Fig. 3a). The BLI-assisted AS-MS approach was utilized to perform the affinity selection, with biotinylated menin protein immobilized onto the streptavidin-coated biosensor and sampled in the synthetic peptide library, and the 12ca5 protein was used as a control. After nLC-MS/MS analysis, six peptides that match the library design were found enriched specifically towards the menin protein when compared with the control (EICs in Fig. S5–S10†). Remarkably, the assigned sequences of hit **1** and hit **6** differ only at the second amino acid position with cyclobutyl alanine in hit **1** and cyclohexyl alanine in hit **6**, a feature which further improved the confidence of the sequence assignment. The six identified amino acid sequences were individually synthesized and their binding was validated against recombinant menin protein. We found the apparent dissociation constants (*K*_D_) of hits **1** and **6** to be ∼100-fold lower than the original sequence (34 and 40 nM *vs.* 3800 nM, respectively). The other four peptides either displayed no binding or had significantly reduced affinity (Fig. 3b).

**Fig. 4:**
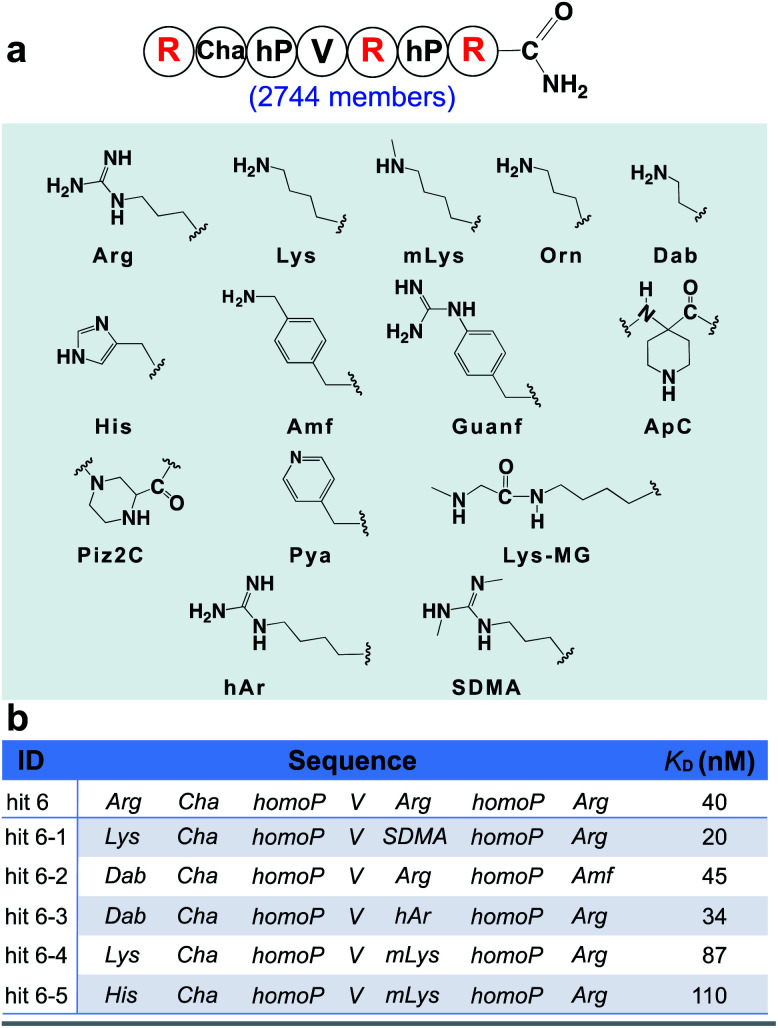
BLI-assisted AS-MS optimizes the positively charged residues of hit 6. (a) Library design based on the sequence of hit 6. The three arginine residues highlighted in red were randomized with the 14 basic amino acid monomers listed. Abbreviations: Arg, l-arginine; Lys, l-lysine; mLys, *N*ε-methyl-l-lysine; Orn, l-ornithine; Dab, L-2,4-diaminobutyric acid; His, l-histidine; Amf, 4-(amino-methyl)-l-phenylalanine; Guanf, 4-guanidino-l-phenylalanine; ApC, 1-piperidine-4-amino-4-caboxylic acid; Piz2C, piperazine-2-caboxylic acid; Pya, 3-(4′-pyridyl)-l-alanine; Lys-MG, l-lysine coupled with *N*-methylglycine; hAr, l-homoarginine; SDMA, l-symmetric-dimethylarginine. (b) Sequences and measured binding affinities towards menin of the top five library hits, see Fig. S17† for experimental details.

From the identified hits in Fig. 3, we observed the positively charged arginine was not swapped for citrulline at any position. This result implies that the positive charge character is more important for binding than the hydrogen bonding character at these positions. Thus, we hypothesized that other positively charged non-canonical monomers could be used to improve the affinity of the peptidomimetic binder and designed the second focused library accordingly, varying the three arginine sites with both canonical basic amino acids (histidine and lysine) and 11 other positively charged non-canonical side chains (Fig. 4a). A similar BLI-assisted AS-MS experiment was performed on this library. After nLC-MS/MS analysis and sequence decoding, five ions were found enriched towards the menin protein compared with the control (EICs in Fig. S11–S15†) and the assigned amino acid sequences were individually synthesized and purified for affinity measurements. We found that the five hits maintain low-nanomolar binding affinity to menin with hit **6-1** further boosting the binding strength by two-fold (apparent dissociation constant, *K*_D_ = 20 nM, Fig. 4b). Interestingly, the canonical arginine or its close derivatives still dominate at both the C-terminus and the middle positions. Methylated lysine can be tolerated at the middle position, but presented slightly decreased binding affinities (Fig. 4b, hit **6-4** and **6-5**). The N-terminus prefers either lysine or diaminobutyric acid residues, which provide an enhanced binding (Fig. 4b, hit **6-1**, **6-2**, and **6-3**).

The BLI-assisted AS-MS platform was thus able to rapidly optimize a menin-binding motif through library design and screening, and generated a series of potent menin-binding peptides by incorporating non-canonical side chains. Of note, the menin-binding peptides reported in this study average 900 Da in molecular mass, and to our knowledge represent the smallest menin peptidomimetic binders. These studies provide promising lead compounds for future pre-clinical investigations and, likewise, potential bioavailable peptide drug development leads to treat MLL-rearranged leukemia.

### Parallel selection demonstrates high selectivity

**Fig. 5:**
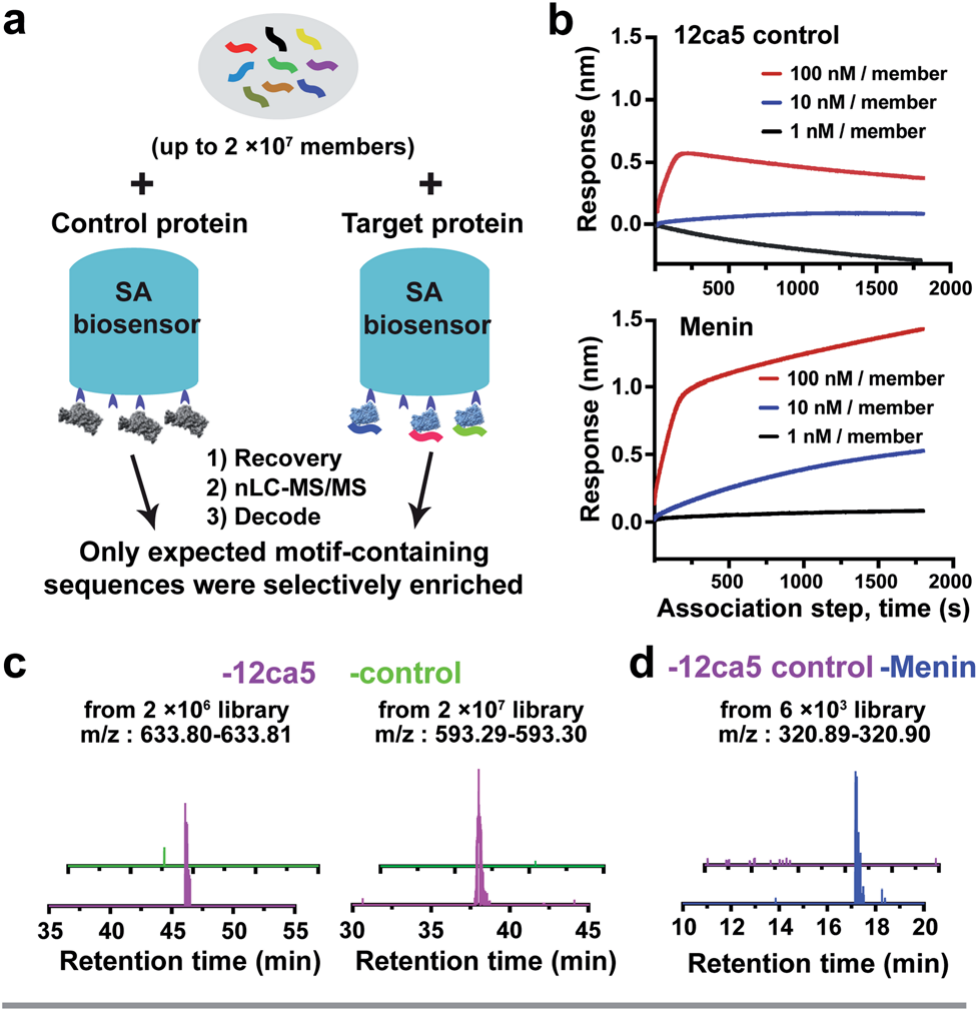
BLI-assisted AS-MS is a novel peptide library screening platform that features real-time monitoring and low background. (a) Workflow of the parallel selection using BLI-assisted AS-MS. (b) The association step of a library screening experiment against menin can be visualized in comparison with the parallel 12ca5 control runs. (c) Extracted ion chromatograms (EICs) demonstrate low or negligible background signals were found in the control protein (ACE2) runs for the *de novo* randomized library screening. (d) EIC of a typical ion enrichment from the focused library screening against menin, with 12ca5 protein as a control.

Non-specific binding was identified as a significant problem during prior affinity selection studies and reducing the background can be a challenging task.^35^ Here, with our newly developed BLI-assisted AS-MS approach, we observed low non-specific binding background in our target protein selection compared with the control protein (Fig. 5a). We believe this enhanced target selectivity may be related to the optimization of BLI commercial systems to minimize non-specific associations. For example, in our single binder ‘capture and release’ experiments, insufficient amount of peptides were recovered for sequence decoding with the control biosensors at peptide concentrations as high as 1.0 μM, whereas target-specific peptides can be recovered efficiently at peptide concentrations as low as 1 nM (Fig. 2b). A similar outcome was obtained when capturing menin single peptide binders using menin-loaded biosensors (Fig. S4†). Moreover, the ion intensities of all peptides of interest were found at background-level (EICs < 10^4^) in all control groups from our randomized library screening, and in contrast, all the peptides recovered and decoded in the 12ca5 protein-specific groups contain the expected HA-binding motif (Fig. 2d and 5c), indicating an enhanced true-positive enrichment. Moving from randomized libraries to focused libraries, target-specific ion enrichment is retained while screening against menin (Fig. 5d). This evidence illustrates that BLI-assisted AS-MS can provide high selectivity toward peptidomimetic binder enrichment (Fig. 5a).

### BLI-assisted AS-MS provides real-time monitoring of the focused library screening process

Bio-Layer Interferometry (BLI) is an optical label-free technology developed for biomolecular interaction measurements with the interference patterns measured in real-time. To benefit from this advantage, we tested and optimized our screening conditions, including the peptide library concentrations and the blocking buffer conditions (detailed protocol in ESI Section 5.1†). Here, using the optimized conditions in our focused library screening, we observed distinct association patterns when comparing the target protein runs with control groups (Fig. 5b). When specific protein–peptide interaction occurs, the association pattern steadily increases as the incubation continues. In comparison, the control run at the highest concentration (100 nM per peptide, 0.1–1.0 mM total library) had an initial association jump but appeared to decrease afterward. Thus, the BLI-assisted AS-MS approach can efficiently provide real-time monitoring of specific protein–peptide interactions during a focused library screening process in direct comparison with a parallel control.

## Discussion

In this study, a novel automated bio-layer interferometry (BLI)-assisted affinity selection-mass spectrometry (AS-MS) approach was developed for rapid and high-throughput peptide library screening. The BLI-assisted AS-MS not only provides real-time surveillance of the library association step but also demonstrates low nonspecific ion capture in the background. Other important benefits of using the BLI-assisted AS-MS include rapid high-throughput screening and computer-controlled peptide capture and release, and these advantages enable high reproducibility and tunability. A typical library screening process using BLI-assisted AS-MS takes no longer than 30 minutes. Conventional labor-intensive library screening is usually subject to experimental variations. In contrast, software-driven library screening using our approach offers high consistency with minimal errors.

We demonstrated the affinity maturation of a known peptide binder using the BLI-assisted AS-MS by incorporating non-canonical amino acids. These unnatural side-chains can greatly expand the breadth of the chemical space surveyed. Although multiple efforts have demonstrated the feasibility of incorporating non-canonical amino acids using phage^38^ or mRNA displays,^39^ routinely accessing non-canonical functionalities in peptide binders to biomolecules remains a limited capability. In comparison, using in-solution AS-MS to screen entirely synthetic libraries can efficiently exploit the advantages of incorporating large numbers of non-canonical amino acids to boost affinity and enhance stability. As illustrated in our study, BLI-assisted AS-MS inherits the full merits of traditional AS-MS and also is amenable to provide a rapid optimization with the capability of accessing both canonical and non-canonical amino acids.

The commercially optimized streptavidin-coated biosensors were used for protein immobilization. Through the single HA tag capture and release against the 12ca5 protein, we demonstrated that one single biosensor tip could provide sufficient ion enrichment for nLC-MS/MS sequencing. Our *de novo* library screening against 12ca5 protein showed that one protein-specific binder was enriched from a synthetic library containing 2 million peptides and three binders were enriched from a 20 million-membered library, beyond which insufficient enrichment resulted in difficulties of binder identification, potentially due to the limited binding capacity of the biosensor tip. However, since our approach is rapid and high-throughput, we envision that it is practical to further increase the library diversity, if needed, by pooling multiple aliquots, as demonstrated previously.^35^ For example, running five 20-million library screenings in parallel is equivalent to screening against one library containing 100-million members.

BLI has found useful applications to studying protein–protein interactions,^33,40^ ligand binding measurement^41^ and detection,^32,42,43^ kinetic analysis^44,45^ and vaccine titer determination.^46^ However, to our knowledge, this report is the first application of bio-layer interferometry to peptide library selections.

Other mass spectrometry techniques, such as matrix-assisted laser desorption/ionization (MALDI-TOF) and surface-enhanced laser desorption/ionization time-of-flight (SELDI-TOF) also allow for differentiation and classification of samples from complex biological mixtures.^47,48^ To enable high sensitivity detection, we coupled affinity selection with nanoscale liquid chromatography-tandem mass spectrometry (nLC-MS/MS), which is able to detect molecules at quantities as low as one femtomole.^49^ Notably, the surface-enhanced affinity capture (SEAC) developed using SELDI technology enables a direct detection of binders from an affinity chip without the need of prior release. Nonspecific absorption and low reproducibility, among several other limitations, hindered the continued development of this method in recent years, however.^50^ In contrast, the BLI-assisted AS-MS approach reported in this study demonstrates high reproducibility, detection sensitivity, binder specificity, and ease of manipulation using an automated workflow.

## Conclusions

Collectively, we developed an automated bio-layer interferometry-assisted affinity selection-mass spectrometry (AS-MS) approach for rapid and high-throughput peptide library screening. Using this approach, we discovered motif-containing peptide binders to the anti-hemagglutinin antibody, 12ca5, from up to 20 million-membered randomized libraries. Similarly, the binding affinity of a sequence derived from the menin binding motif was improved by approximately two hundred-fold upon screening two focused synthetic peptide libraries with a diversity of thousands of members. The BLI-assisted AS-MS approach reported in this study offers several advantageous features over existing AS-MS methods such as programmable control, high specificity, high-throughput, high reproducibility, low background, and real-time monitoring. Although *de novo* discovery of target-specific peptide binders and rapid optimization of known binders are still challenging in the field, we believe our BLI-assisted AS-MS platform could provide an important contribution to facilitate such efforts.

## Data availability

All the data supporting this article have been included in the main text and the ESI.†

## Author contributions

G. Z., C. L., A. J. Q., and B. L. P. designed research; G. Z., C. L. and A. J. Q. performed experiments, with input from A. L. and B. L. P.; G. Z., C. L., A. L. and A. J. Q. analyzed data; G. Z., and C. L. generated figures; and G. Z. wrote the manuscript with input from all authors.

## Conflicts of interest

B. L. P. is a co-founder of Amide Technologies and Resolute Bio. Both companies focus on the development of protein and peptide therapeutics.

## Supplementary Material

SC-012-D1SC02587B-s001
