# Supplementary material for: Automated affinity selection for rapid discovery of peptide binders
Source: Chem Sci. 2021 Jul 14;12(32):10817–24. doi: 10.1039/d1sc02587b (PMC8372318; doi:10.1039/d1sc02587b)
Supplement: SC-012-D1SC02587B-s001 [file SC-012-D1SC02587B-s001.pdf]

Electronic Supplementary Material (ESI) for Chemical Science.
This journal is © The Royal Society of Chemistry 2021

Electronic Supplementary Information for

Automated Affinity Selection for Rapid Discovery of Peptide Binders

Genwei Zhang[1], Chengxi Li[1], Anthony J. Quartararo[1], Andrei Loas[1], Bradley L. Pentelute[1-4, *]

[1]*Massachusetts Institute of Technology, Department of Chemistry, 77 Massachusetts Avenue, Cambridge, MA 02139, USA.*

[2]*The Koch Institute for Integrative Cancer Research, Massachusetts Institute of Technology, 500 Main Street, Cambridge, MA 02142, USA.*

[3]*Center for Environmental Health Sciences, Massachusetts Institute of Technology, 77 Massachusetts Avenue, Cambridge, MA 02139, USA.*

[4]*Broad Institute of MIT and Harvard, 415 Main Street, Cambridge, MA 02142, USA.*

[*]*To whom correspondence should be addressed: blp@mit.edu*

Table of Contents

1. MATERIALS AND METHODS 4
1.1 MATERIALS 4
1.2 MANUAL FAST-FLOW SYNTHESIS OF CANONICAL PEPTIDES 4
1.3 MANUAL SOLID-PHASE SYNTHESIS OF NON-CANONICAL PEPTIDES 5
1.4 PEPTIDE CLEAVAGE 5
1.5 PROTEIN PREPARATION 5
1.5.1 Menin expression and purification 5
1.5.2 Protein biotinylation 6
1.6 PEPTIDE PURIFICATION 6
1.6.1 Reverse-Phase HPLC 6
1.6.2 Flash chromatography purification 6
1.7 LC-MS ANALYSIS 6
1.8 SPLIT-AND-POOL SYNTHESIS OF PEPTIDE LIBRARIES 7
1.9 SOLID PHASE EXTRACTION 7
1.10 CAPTURE OF CONTROL PEPTIDE BINDERS USING STREPTAVIDIN-COATED BIOSENSOR 8
1.11 BLI-ASSISTED AFFINITY SELECTION FROM RANDOM LIBRARIES 8
1.12 NANO LC-MS/MS (NLC-MS/MS) SEQUENCING 8
1.13 BIO-LAYER INTERFEROMETRY 9
2. PROTEIN PURITY DETERMINATION 9
2.1 BIOTINYLATED 12CA5 PROTEIN 9
2.2 PURIFIED MENIN PROTEIN 10
3. RAW LC-MS DATA 11
3.1 LC-MS TRACES OF PURIFIED HA TAG 11
3.2 LC-MS TRACES OF PURIFIED MBM1 AND ITS TRUNCATION VARIANTS 12
3.2.1 Biotin-MBM1 12
3.2.2 Biotin-tMBM1.0 13
3.2.3 Biotin-tMBM1.1 14
3.2.4 Biotin-tMBM1.2 15
3.2.5 Biotin-tMBM1.3 16
3.3 LC-MS TRACES OF PURIFIED LIBRARY SCREEN HITS 17
3.3.1 Biotin-hit 1 through 6 17
3.3.2 Biotin-hit 6-1 through 6-5 23
4. LIBRARY PREPARATION AND QC 28
4.1 THREE RANDOMIZED LIBRARIES 28
4.2 FOCUSED LIBRARIES 1 AND 2 28
5. BLI-ASSISTED LIBRARY SCREEN AND AFFINITY ENRICHMENT 29
5.1 PROCEDURES OF BLI-ASSISTED LIBRARY SCREEN 29

5.2 Indirect capture of HA tag using BLI AS-MS 32
5.3 Recovery of tMBM1.0 sequence using BLI AS-MS 33
5.4 Extracted ions of enriched peptide from library 1 34
5.5 Extracted ions of enriched peptide from library 2 40
6. BINDING AFFINITY MEASUREMENT OF LIBRARY SCREEN HITS USING BLI 45
7. REFERENCES 47

1. Materials and Methods

1.1 Materials

Unless otherwise noted, all chemicals and reagents were obtained from commercial sources without further purification and details can be found below. *N*,*N*-Diisopropylethylamine (DIEA) was obtained from a Seca Solvent Purification System by Pure Process Technology.

H-Rink Amide-ChemMatrix resin was obtained from PCAS BioMatrix. Tentagel M NH2 resin was obtained from Rapp Polymere. 4-[(*R*,*S*)-α-[1-(9*H*-Fluoren-9-yl)-methoxyformamido]-2,4-dimethoxybenzyl]-phenoxyacetic acid (Fmoc-Rink amide linker) was obtained from Chem-Impex International. Biotin-PEG$_4$-propionic acid and Biotin-PEG$_4$-NHS were both purchased from ChemPep Inc. Peptide synthesis-grade *N*, *N*-dimethylformamide (DMF), dichloromethane (DCM), diethyl ether, and HPLC-grade acetonitrile were obtained from VWR International. 1-[Bis(dimethylamino)methylene]-1*H*-1,2,3-triazolo[4,5-b]pyridinium-3-oxid-hexafluorophosphate (HATU) was purchased from P3 BioSystems. Trifluoroacetic acid (TFA; for HPLC, ≥99%), piperidine (ReagentPlus; 99%), triisopropylsilane (98%), 1,2-ethanedithiol (≥98%), phenylsilane (97%) and tetrakis(triphenylphosphine)palladium(0) (99%), were purchased from MilliporeSigma. Biotechnology grade Bovine Serum Albumin (BSA) was obtained from VWR International. Ultrapure water was obtained by filtering deionized water with a Milli-Q water purification system (Millipore). Fmoc-Ala-OH, Fmoc-Arg(Pbf)-OH, Fmoc-Asn(Trt)-OH, Fmoc-Asp(tBu)-OH, Fmoc-Gln(Trt)-OH, Fmoc-Glu(tBu)-OH, Fmoc-Gly-OH, Fmoc-His(Trt)-OH, Fmoc-Leu-OH, Fmoc-Lys(Boc)-OH, Fmoc-Met-OH, Fmoc-Phe-OH, Fmoc-Pro-OH, Fmoc-Ser(tBu)-OH, Fmoc-Thr(tBu)-OH, Fmoc-Trp(Boc)-OH, Fmoc-Tyr(tBu)-OH, and Fmoc-Val-OH were purchased from Advanced ChemTech (Louisville, KY). Fmoc-4-fluoro-L-phenylalanine, Fmoc-3,4-difluoro-L-phenylalanine, Fmoc-3,4,5-trifluoro-L-phenylalanine, Fmoc-pentafluoro-L-phenylalanine, Fmoc-4-(Boc-amino)-L-phenylalanine, Fmoc-2-trifluoromethyl-L-phenylalanine, Fmoc-3-methoxy-L-phenlyalanine, Fmoc-4-cyano-L-phenylalanine, Fmoc-4-nitro-L-phenylalanine, Fmoc-3,4-dimethoxy-L-phenylalanine, Fmoc-L-phenylglycine, Fmoc-L-homophenylalanine, Fmoc-β-cyclohexyl-L-alanine, Fmoc-β-cyclobutyl-L-alanine, Fmoc-β-cyclopropyl-L-alanine, (S)-2-Fmoc-amino heptanoic acid, Fmoc-3-(4'-pyridyl)-L-alanine, Fmoc-3-(2-naphthyl)-L-alanine, Fmoc-cis-4-fluoro-Pro-OH, Fmoc-L-Homo-Pro-OH, Fmoc-L-Thia-Pro-OH, Fmoc-Aib-OH, Fmoc-L-α-tert-butylglycine, Fmoc-L-citrulline-OH, Fmoc-L-ornithine(Boc)-OH, Nα-Fmoc-Nγ-Boc-L-2,4-diaminobutyric acid, Fmoc-4-(Boc-amino-methyl)-L-phenylalanine, 1-Boc-piperidine-4-Fmoc-amino-4-carboxylic acid, Fmoc-piperazine(boc)-2-carboxylic acid, Fmoc-3-(4'-pyridyl)-L-alanine, Fmoc-L-Lys(Alloc), Fmoc-L-homoarginine(Pbf) were all purchased from Chem-Impex International. Fmoc-L-Lys(Me,Boc)-OH, Fmoc-Phe(4-Boc$_2$-guanidino)-OH and Fmoc-SDMA(Boc$_2$)-ONa were purchased from Sigma-Aldrich.

1.2 Manual fast-flow synthesis of canonical peptides

Peptide synthesis was carried out with a manual fast-flow synthesizer[1] on a 0.075 mmol scale, using H-Rink Amide-ChemMatrix resin (loading capacity 0.49 mmol/g). A typical coupling cycle includes: 1) a 30 s coupling with a mixture of Fmoc-protected amino acid (1 mmol), HATU

(0.95 mmol), and DIEA (2.9 mmol, 500 µL) in 2.5 mL of DMF, at a flow rate of 6 mL/min; 2) 1 min amine-free DMF wash, at a flow rate of 20 mL/min; 3) 20 sec deprotection with 20% (v/v) piperidine in DMF, at a flow rate of 20 mL/min followed by another 1 min DMF wash, at a flow rate of 20 mL/min. Reagents and solvents were delivered to a reusable stainless steel reactor[1] containing the resin by either an HPLC pump (DMF or 20% (v/v) piperidine in DMF) or a syringe pump (mixture of Fmoc-protected amino acid, HATU and DIEA). The reactor temperature was maintained at 70 °C in a water bath duration the entire synthesis. After each synthesis completion, resins were retrieved from the reactor and washed with DCM (5x) and dried in a desiccator with reduced pressure.

1.3 Manual solid-phase synthesis of non-canonical peptides

Non-canonical amino-acid containing peptides were synthesized manually in batch by standard solid-phase protocols, typically at the 0.05 mmol scale on H-Rink Amide-ChemMatrix resin within Torviq syringes (size 6 mL). Briefly, resins were soaked with DMF for 15 min before starting the peptide sequence assembly. Coupling steps were performed under room temperature for 20 min with Fmoc-protected amino acids (0.5 mmol, 10 equivalents) dissolved into 1.25 mL HATU (0.38 M solution in DMF, 9.5 equivalents) and 250 µL DIEA for activation (3 equivalents). The resin was stirred several times during coupling then washed (5×) with DMF before deprotection with 20% (v/v) piperidine in DMF (2× with 5 min each time), and finally washed again (5×) with DMF to finish the cycle. After completing all peptide couplings, the resin was washed with DMF (5×) and DCM (5×) sequentially, and then dried under reduced pressure before proceeding to cleavage.

1.4 Peptide cleavage

Peptides were cleaved from the resin and all side chains were deprotected simultaneously with 2.5% (v/v) 1,2-ethanedithiol (EDT), 5% (v/v) water, 5% (v/v) phenol, 5% (v/v) thioanisole in neat TFA for 1.5-2.0 h at room temperature. Five mL of cleavage cocktail was used for 0.1 mmol of the peptide. The resulting cleaved solution was washed with dry ice-cold ether and centrifuged at 4,000 rpm for 3 min to precipitate the crude peptides. The obtained solids were dissolved in water/acetonitrile (50:50) and lyophilized.

1.5 Protein preparation

The anti-hemagglutinin monoclonal antibody (also called ‘12ca5’) was commercially available; recombinant menin protein was produced in-house in bacteria, and both 12ca5 and menin proteins were then biotinylated for BLI capture experiments. The concentrations of menin and 12ca5 proteins were determined using both UV absorbance under 280 nm (using calculated extinction coefficient) and the Bradford method (using BSA as the standard).

1.5.1 Menin expression and purification

The complete MEN1 coding sequence was PCR-amplified with pre-designed primers (forward primer: 5’-ATGGGGCTGAAGGCCGCCCA-3’, reverse primer: 5’-TCAGAGGCCTTTGCGCTGC-3’) from a commercially available plasmid as the template. The PCR product (with an adenine nucleotide acid overhand at the 3’-end) was mixed with

PET/SUMO plasmid for ligation, into a six consecutive histidine expression plasmid and transformed into *Escherichia coli* DH5α bacteria. The sequence-verified plasmid construct was subsequently transformed into *E. coli* BL21 (DE3) strain for protein production. Half mM isopropyl 1-thio-β-D-galactopyranoside (IPTG) was used to induce gene expression in *E. coli* BL21 transformants. Cells were lysed in lysis buffer (50 mM Tris·HCl, 150 mM NaCl pH 7.5, supplemented with protease inhibitor, 5 μg/mL DNase, and 0.5 mg/mL lysozyme) for 2-4 h at 4 °C. Recombinant menin was purified on a prepacked Ni-NTA column (PROTEINDEX™ HiBond™ Ni-NTA Agarose 6FF, Marvelgent Biosciences) following the vendor instructions. 20 mL of *E. coli* lysate was collected from 500 mL of culture, and the amount of recombinant menin was determined as ~4.0 mg according to the band intensity on a 10% Sodium Dodecyl Sulfate Poly-Acrylamide Gel Electrophoresis (SDS-PAGE) gel comparing to standard markers. In the end, 0.5 mg of purified menin was obtained with a recovery rate of ~12.5%. SDS-PAGE and Coomassie Brilliant Blue staining confirmed the purity of recombinant menin.

1.5.2 Protein biotinylation

Biotin-PEG_4-NHS (Chempep, catalog #271611) was used for protein biotinylation. Two equivalents of Biotin-PEG_4-NHS was mixed with the protein of interest in 1 × PBS (with 5% DMF) and the coupling reaction was carried out overnight at 4 °C. After coupling, excess Biotin-PEG_4-NHS was removed by spin filtration with an appropriate molecular weight cut-off (30K for menin and 50K for 12ca5) and the buffer was changed to 1 × PBS.

1.6 Peptide purification

Peptides with crude purity >85% were purified using a Biotage Selekt© flash purification system and peptides with crude purity <85% were purified using reverse-phase HPLC. Both methods and detailed information can be found below.

1.6.1 Reverse-Phase HPLC

Crude peptides with purity below 80% were dissolved in water with 0.1% TFA and then purified by semipreparative reverse-phase HPLC using a Waters 600 HPLC system (Agilent Zorbax SB-C3 column: 9.4 × 250 mm, 5 μm; or Agilent Zorbax SB-C18 column: 9.4 × 250 mm, 5 μm). The gradient used was 10 to 61% acetonitrile over 60 min with a 4.0 mL/min flow rate. HPLC fractions were analyzed by LC–MS and pure fractions were combined and lyophilized.

1.6.2 Flash chromatography purification

Synthetic peptides with the estimated LC-MS crude purity above 85% were purified with a Biotage Selekt© instrument using Biotage Sfär C18 D column (Duo 100 Å 30 μm, 12 g) under a 5 to 60% acetonitrile gradient over 12 column volumes, and the flow rate was set to 12 mL/min. Fractions were collected based on absorbance at 214 nm and then subjected to LC-MS analysis before combining the pure fractions and lyophilization.

1.7 LC-MS analysis

Crude synthetic peptides or peptide fractions from the purification steps were analyzed by LC-MS (Agilent 6545 or 6550 ESI Q-TOF) using an Agilent Zorbax 300SB-C3 or Phenomenex

Luna C18 column. Total ion chromatograms and integrated MS over the main peak are provided in Section 4.

Condition 1: Analysis was performed on an Agilent 1290 Infinity HPLC coupled to an Agilent 6545 ESI-Q-TOF mass spectrometer. MS was run in positive ionization mode, extended dynamic range (2 GHz), and standard mass range (m/z in the range of 300 to 3000 a.m.u.). The solvent mixtures used for LC-MS chromatography were: A = water + 0.1% formic acid (LC-MS grade), B = acetonitrile + 0.1% formic acid (LC-MS grade). The following conditions were used for peptide analysis. Column: Zorbax 300-SB C3 (5 μm, 150 x 2.1 mm, 300 Å silica); Flow Rate: 0.8 mL/min; Gradient: 1% B 0-2 min, linearly ramp from 1% B to 61% B 2 to 11 min, 61% B to 95% B 11 to 12 min. Post time is 1% B for 3 min. MS data were acquired from 4 to 11 minutes.

Condition 2: Analysis was performed on an Agilent 1290 Infinity HPLC coupled to an Agilent 6550 Q-TOF with Dual Jet Stream ESI ionization and iFunnel. MS was run in positive ionization mode, extended dynamic range (2 GHz), and low mass range (m/z in the range of 100 to 1700 a.m.u.). The solvent mixtures used were as above. Column: Phenomenex Luna C18 (3 μm, 150 x 1 mm, 100 Å silica); Flow Rate: 0.5 mL/min; Gradient: 1% B 0-2 min, linearly ramp from 1% B to 61% B 2 to 14 min, 61% B. Post time is 1% B for 3 min. MS data were acquired from 4 to 14 minutes.

1.8 Split-and-pool synthesis of peptide libraries

Split-and-pool synthesis was carried out on a 30 μm TentaGel resin (0.26 mmol/g) for 10^8-member library. Each coupling step was carried out as follows: solutions of Fmoc-protected amino acids, HATU (0.38 M in DMF; 0.9 equiv. relative to amino acid), and DIEA (1.1 equiv. for histidine; 3 equiv. for all other amino acids) were each added to individual portions of resin. Couplings were allowed to proceed for 20 min, and resin portions were recombined, and washed with DMF for deprotection. Fmoc removal step was carried out by treatment of the resin with 20% piperidine in DMF (1x flow wash; 2x 5 min batch treatments). Resin was washed again with DMF before splitting. Splits were performed by suspending the resin in DMF and dividing it evenly among 18 plastic fritted syringes on a manifold (for 18 canonical amino acids except isoleucine and cysteine). The cycle was then continued with the next coupling. Portions of the 10^8-member library comprising 2×10^7 and 2×10^6 members were taken prior to cleavage from resin to give three libraries of increasing size from the same split-and-pool synthesis batch.

1.9 Solid phase extraction

Synthetic peptide libraries were cleaned using the solid phase extraction method on a 6 mL C18 cartridge (purchased from Supelco® Discovery grade, DSC-18). A typical solid phase extraction involves four steps: firstly, the cartridge is pre-conditioned with 100% acetonitrile (0.1% TFA) to wet the sorbent and equilibrated with 100% water (0.1% TFA); secondly, the loading solution containing the peptide library is percolated through the solid phase; thirdly, the sorbent is then washed with 100% water (0.1% TFA) to remove impurities; lastly, the library is collected during the elution step with 30% water and 70% acetonitrile (0.1% TFA). Library eluate was then lyophilized and stored at -80 ˚C before use.

1.10 Capture of control peptide binders using streptavidin-coated biosensor

Biotinylated anti-hemagglutinin monoclonal antibody (12ca5) or menin proteins at a concentration of 200 nM were immobilized onto streptavidin-coated biosensor tips (ForteBio, Streptavidin (SA), Dip and Read Biosensors, part no: 18-5021) using a ForteBio Octet RED96 BLI system (Octet RED96) and then sampled into different concentrations (1000, 100, 10, 1, 0.1 nM) of the control peptide binders in a kinetic buffer (K.B.: 1 × PBS with 0.1% BSA and 0.02% tween). The association step was 30 min long with 1,000 rpm agitation. A brief wash (20 s) in the K.B. was given before moving the biosensor tip into 6 M guanidine hydrochloride, 200 mM phosphate, pH 6.8 for elution. The assay temperature was kept constant at 30 °C and the process was monitored in real-time with the ForteBio Biosystems Data Acquisition Software. Recovered eluates were concentrated via C18 ZipTip® pipette tips and lyophilized. Dry powders were resuspended in 6 µL water (0.1% formic acid), and then 5 µL was submitted for nLC-MS/MS analysis (Section 2.12). Biotinylated ACE2 protein was used as a control in a parallel run.

1.11 BLI-assisted affinity selection from random libraries

Similar to the control binder capture (Section 2.10), biotinylated proteins were immobilized onto the SA biosensors and then sampled into randomized libraries in the K.B. A typical association step was 30 min long with 1,000 rpm agitation, and a brief wash (20 s) in the K.B. was given before moving the biosensor tip into 6 M guanidine hydrochloride, 200 mM phosphate, pH 6.8 for elution. The whole screening process was monitored in real-time and the temperature was kept constant at 30 °C. Eluates were concentrated via C18 ZipTip® pipette tips and lyophilized. Dry powders were resuspended in 6 µL water (0.1% formic acid), and then 5 µL were submitted for nLC-MS/MS analysis.

1.12 Nano LC-MS/MS (nLC-MS/MS) sequencing

Peptide sequence analysis was performed on an EASY-nLC 1200 (Thermo Fisher Scientific) nano-liquid chromatography handling system with an Orbitrap Fusion Lumos Tribrid Mass Spectrometer (Thermo Fisher Scientific). Samples prepared from the library screening steps were run on a PepMap RSLC C18 column (2 µm particle size, 15 cm × 50 µm ID; Thermo Fisher Scientific, P/N ES801). A nanoViper Trap Column (C18, 3 µm particle size, 100 Å pore size, 20 mm × 75 µm ID; Thermo Fisher Scientific, P/N 164946) was used for desalting. The standard nano-LC method was run at 40 °C and a flow rate of 300 nL/min with the following gradient: 1% solvent B in solvent A ramping linearly to 61% B in A over 60 or 90 min, where solvent A = water (0.1% FA), and solvent B = 80% acetonitrile, 20% water (0.1% FA). Positive ion spray voltage was set to 2200 V. Orbitrap detection was used for primary MS, with the following parameters: resolution = 120,000; quadrupole isolation; scan range = 200–1400 m/z; RF lens = 30%; AGC target = 1×10^6; maximum injection time = 100 ms; 1 microscan. Secondary MS spectra acquisition was done in a data-dependent manner: dynamic exclusion was employed such that a precursor was excluded for 30 s if it was detected four or more times within 30 s (mass tolerance: 10.00 ppm); monoisotopic precursor selection used to select for peptides; intensity threshold was set to 5×10^4; charge states 2-10 were selected; and precursor selection range was set to 200-1400 m/z. The top 15 most intense precursors that met the preceding

criteria were subjected to subsequent fragmentation. Three fragmentation modes—collision-induced dissociation (CID), higher-energy collisional dissociation (HCD), and electron-transfer/higher-energy collisional dissociation (EThcD)—were used for acquisition of secondary MS spectra. Only precursors with charge states 3 and above were subjected to all three fragmentation modes; precursors with charge states of 2 were subjected to CID and HCD only. For all three modes, detection was performed in the Orbitrap (resolution = 30,000; quadrupole isolation; isolation window = 1.3 m/z; AGC target = 2×10^4; maximum injection time = 100 ms; 1 microscan). For CID and HCD, a collision energy of 30% and 25% was used, respectively. For EthcD, a supplemental activation collision energy of 25% was used.

1.13 Bio-layer interferometry

Individual peptide-binding validation was performed using the ForteBio Octet RED96 BLI system (Octet RED96), with the temperature at 30 °C and agitation speed at 1,000 rpm. In brief, streptavidin (SA) biosensor tips were dipped into 200 μL of 1.0 μM biotinylated peptide solution (in the kinetic buffer (K.B.): 1 × PBS with 0.1% BSA and 0.02% tween) to immobilize the peptides. The tips loaded with peptides were then moved into wells containing various concentrations (1000, 500, 250, 125, 62.5, 31.3, and 15.6 nM) of recombinant menin protein in the K.B. to obtain the association curve. After the 180 s association step, the tips were dipped back into the K.B. to obtain the dissociation curve. Buffer-only and protein-only conditions (at the highest sampled protein concentration) were used as references for background subtraction. The association and dissociation curves were fitted with the ForteBio Biosystems Data Analysis Software under 7 experimental conditions (n = 7, global fitting algorithm, binding model 1:1) to obtain the apparent dissociation constant (K_D).

2. Protein purity determination

2.1 Biotinylated 12ca5 protein

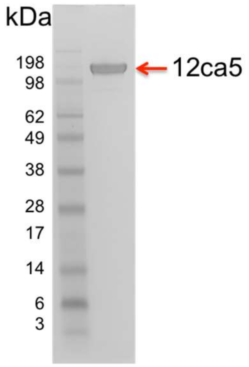

Figure S1. Biotinylated 12ca5 protein (denoted with the red arrow) on a 10% SDS-PAGE gel. This purified protein was used for subsequent library screens.

2.2 Purified menin protein

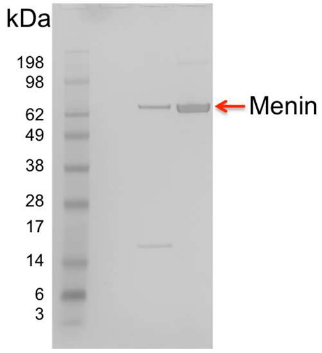

Figure S2. Recombinant menin protein (denoted with the red arrow, on a 10% SDS-PAGE gel) was produced in *E. coli* BL21 strain and purified with a C-terminal His_6 tag. The expected protein size is approximately 63 kDa. The purified protein was used for subsequent library screens and peptide binding affinity characterizations.

3. Raw LC-MS data

Note: After purification, peptide purity was assessed under either **Condition 1** or **Condition 2** in Section **1.7**. Raw LC-MS data is presented as the total ion current chromatograms (TIC), which were analyzed using the software Agilent MassHunter Qualitative Analysis v10.0 for each compound and the MS spectrum was extracted over the main product peaks. The notation -0.98 at the end of each sequence indicates a C-terminal amide group.

3.1 LC-MS traces of purified HA tag

Sequence: YPYDVPDYAK (-0.98)

LC-MS Method: **Condition 1**

Observed: 1228.59 Da Calculated: 1228.58 Da

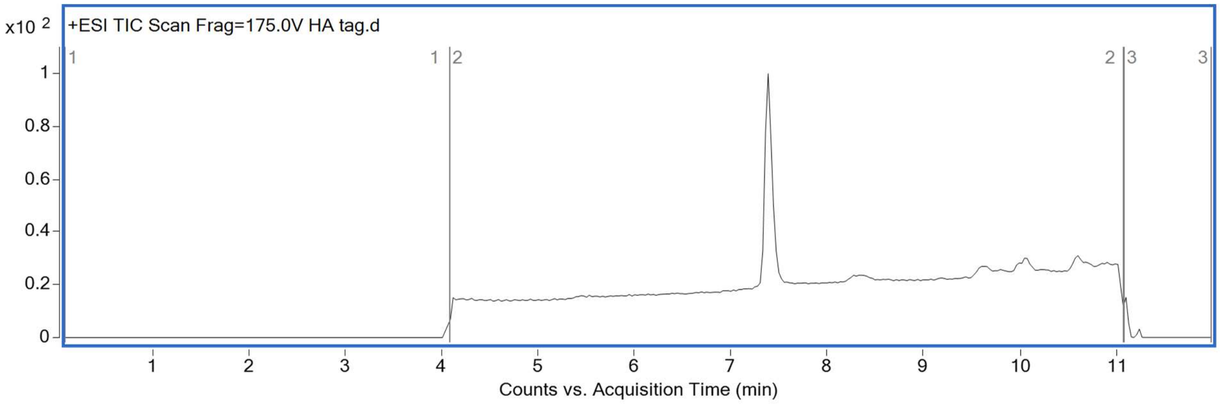

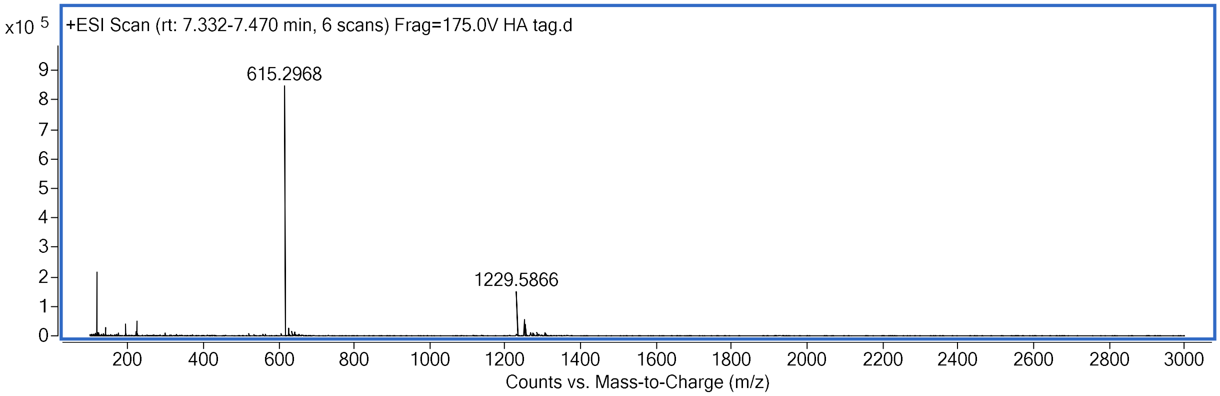

3.2 LC-MS traces of purified MBM1 and its truncation variants

3.2.1 Biotin-MBM1

Sequence: Biotin-$(GS)_4$-SARWRFPARPGT (-0.98)

LC-MS Method: **Condition 1**

Observed: 2202.03 Da   Calculated: 2202.05 Da

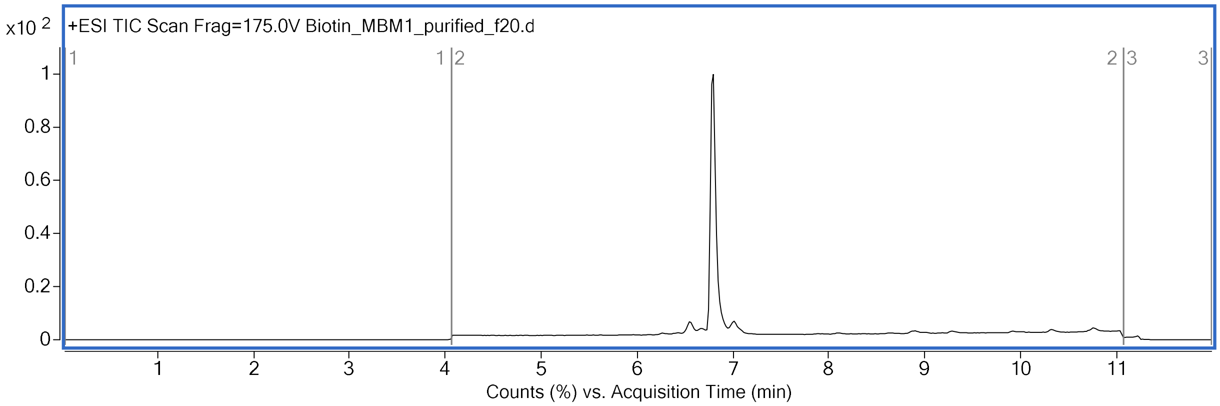

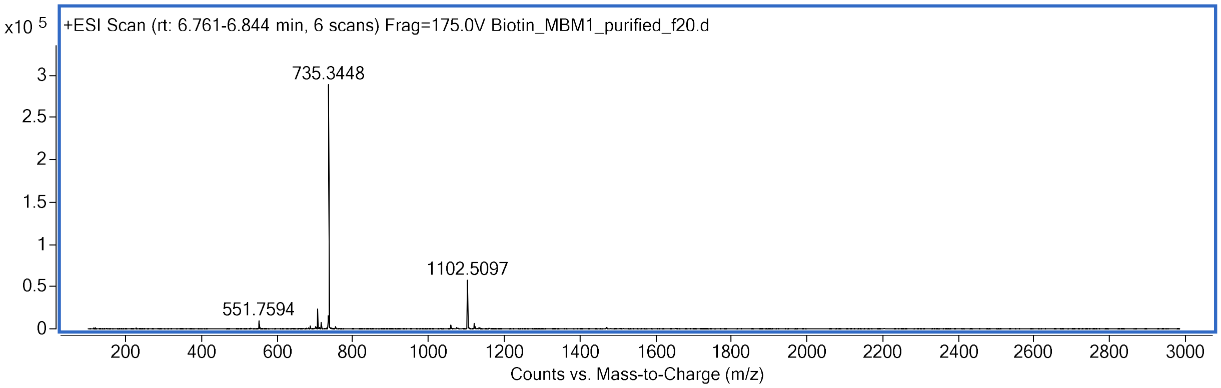

3.2.2 Biotin-tMBM1.0

Sequence: Biotin-(PEG)$_4$-RWRFPARPR (-0.98)

LC-MS Method: **Condition 1**

Observed: 1712.97 Da Calculated: 1712.94 Da

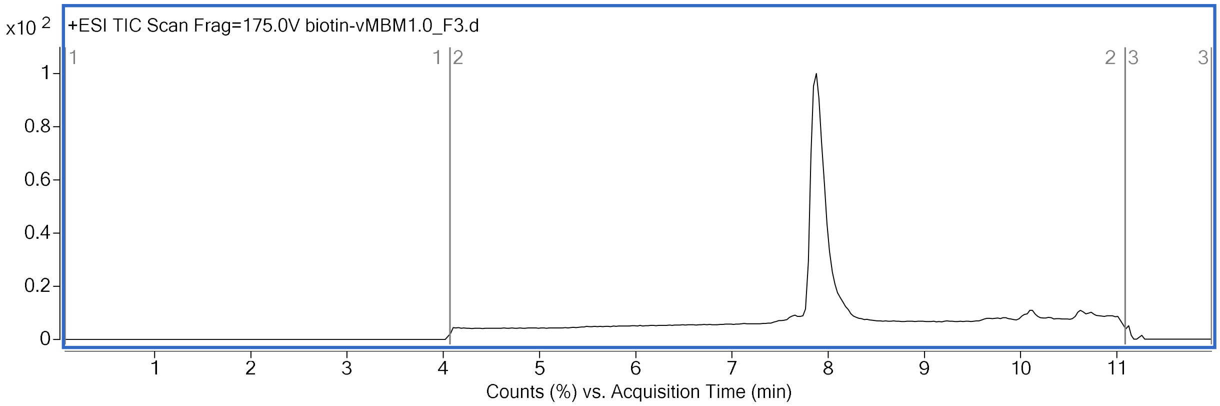

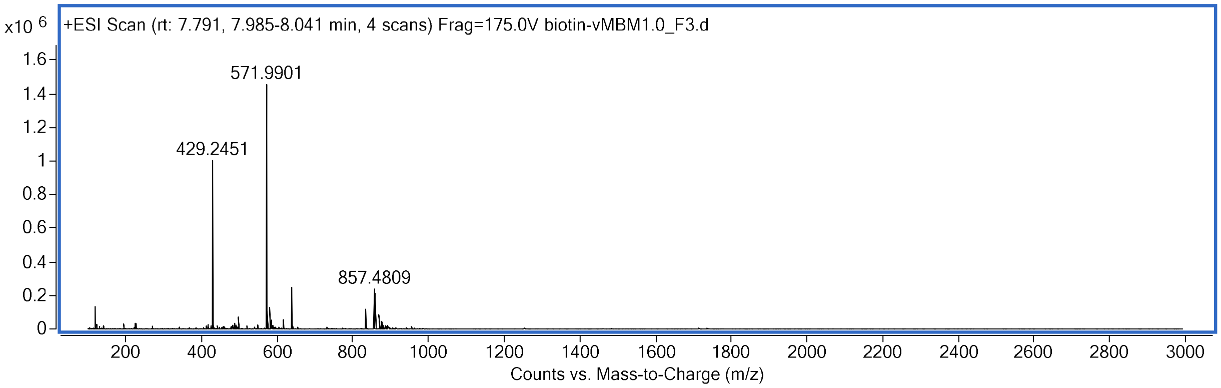

3.2.3 Biotin-tMBM1.1

Sequence: Biotin-(PEG)$_4$-WRFPARPR (-0.98)

LC-MS Method: **Condition 2**

Observed: 1556.85 Da Calculated: 1556.84 Da

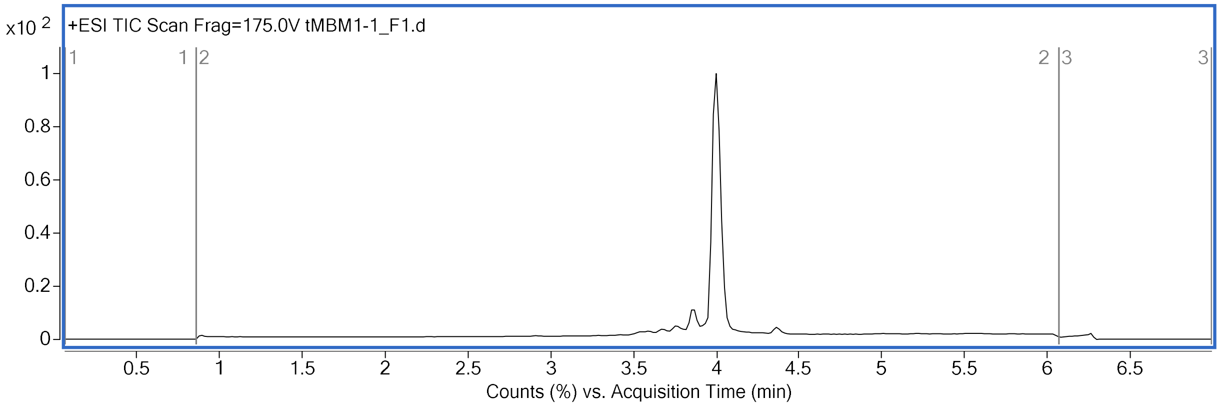

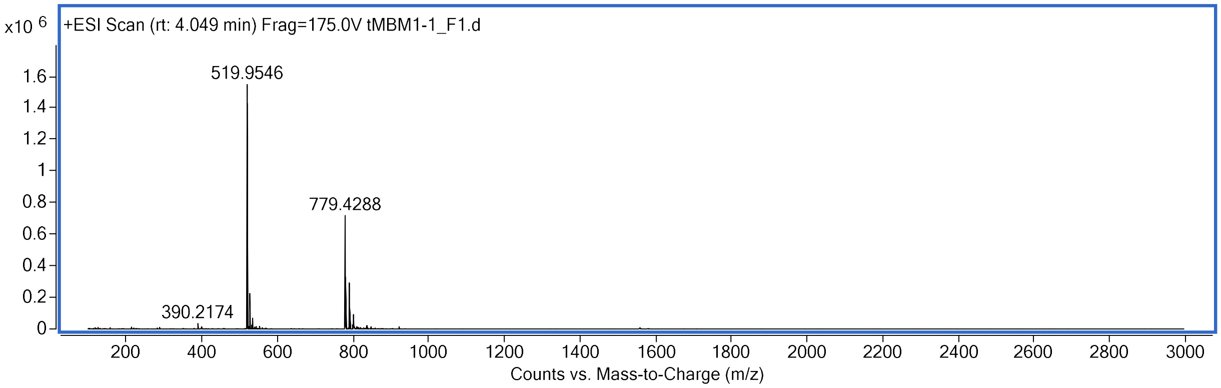

3.2.4 Biotin-tMBM1.2

Sequence: Biotin-$(PEG)_4$-RFPARPR (-0.98)

LC-MS Method: **Condition 2**

Observed: 1370.77 Da Calculated: 1370.76 Da

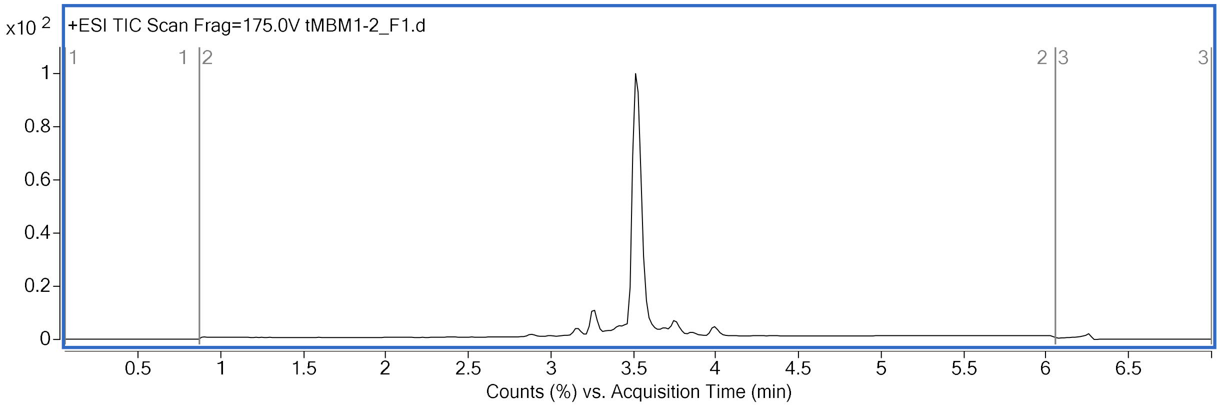

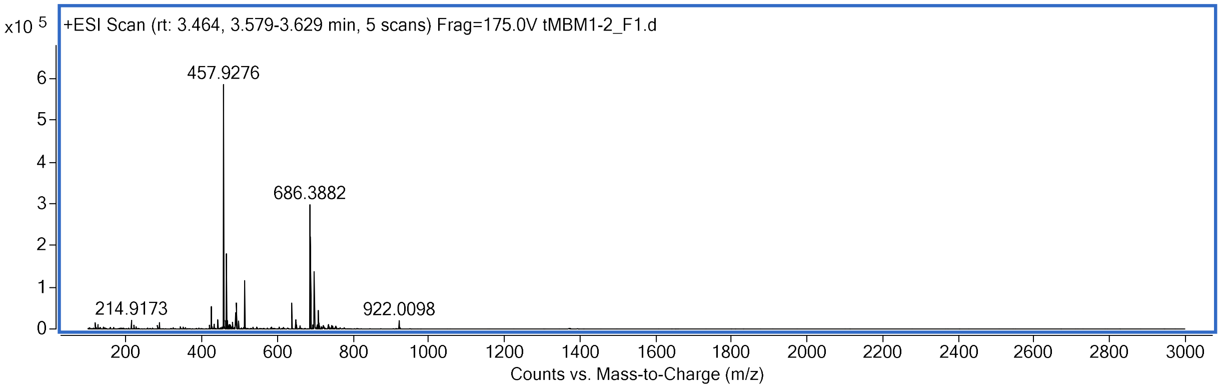

3.2.5 Biotin-tMBM1.3

Sequence: Biotin-(PEG)$_4$-FPARPR (-0.98)

LC-MS Method: **Condition 2**

Observed: 1214.67 Da Calculated: 1214.66 Da

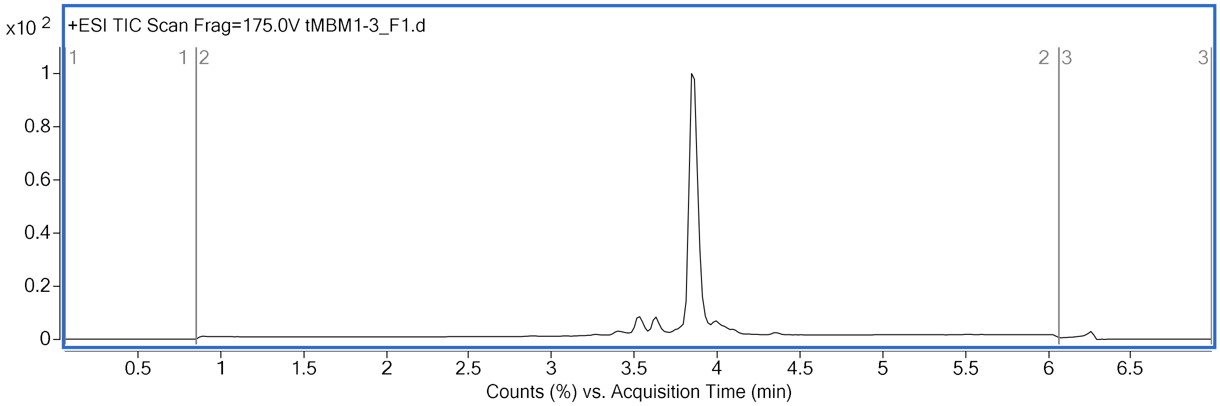

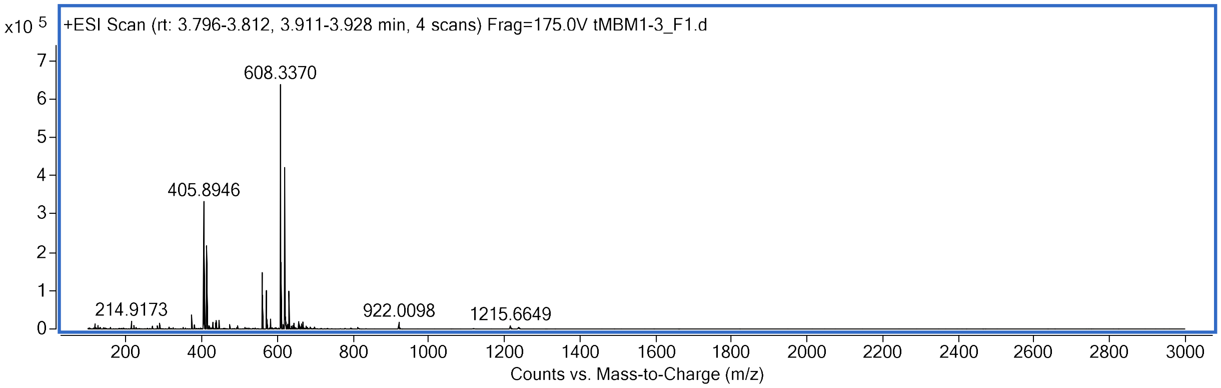

3.3 LC-MS traces of purified library screen hits

3.3.1 Biotin-hit 1 through 6

Biotin-hit 1

Sequence: Biotin-$(PEG)_4$-R(Cba)(homoP)VR(homoP)R (-0.98)

(Cba, β-cyclobutyl-L-alanine; homoP, L-homo-proline)

LC-MS Method: **Condition 2**

Observed: 1404.85 Da		Calculated: 1404.84 Da

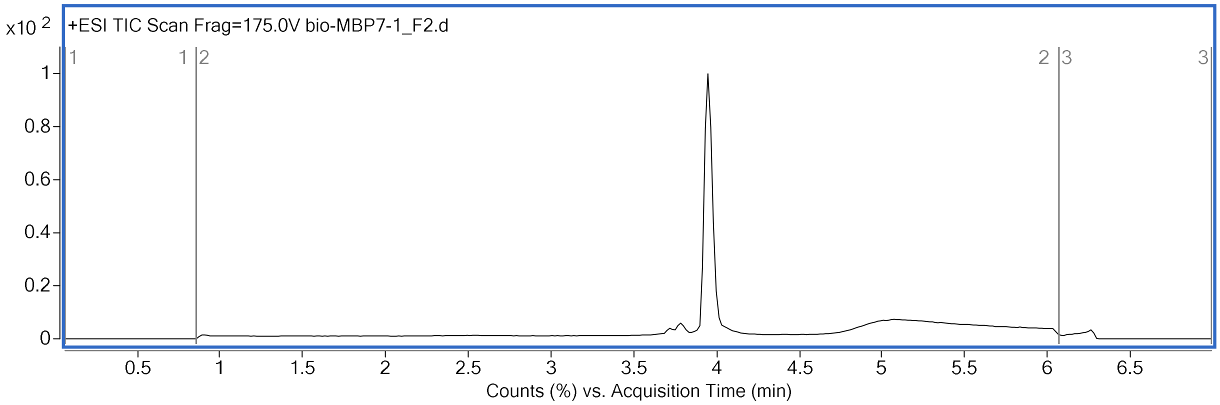

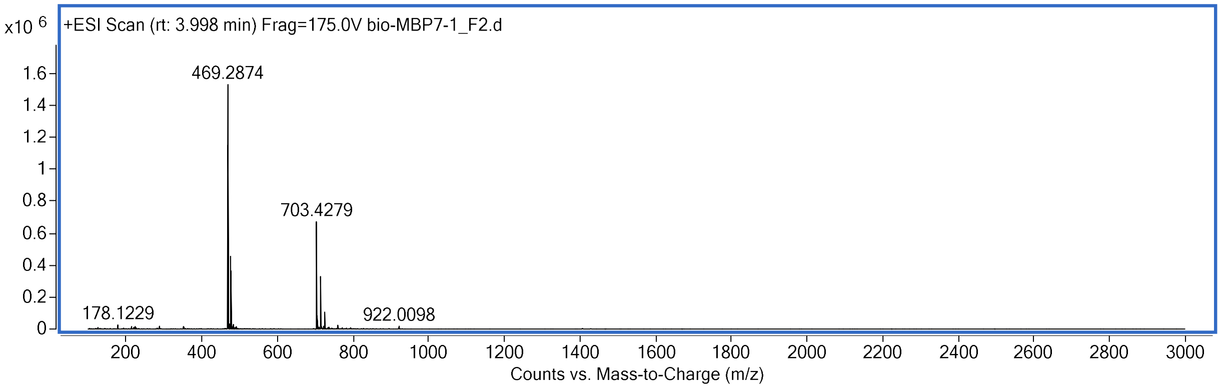

Biotin-hit 2

Sequence: Biotin-(PEG)$_4$-R(homof)P(Tle)R(FP)R (-0.98)

(homof, L-homo-phenylalanine; Tle, L-α-tert-butylglycine; FP, cis-4-fluoro-proline)

LC-MS Method: **Condition 2**

Observed: 1444.82 Da Calculated: 1444.81 Da

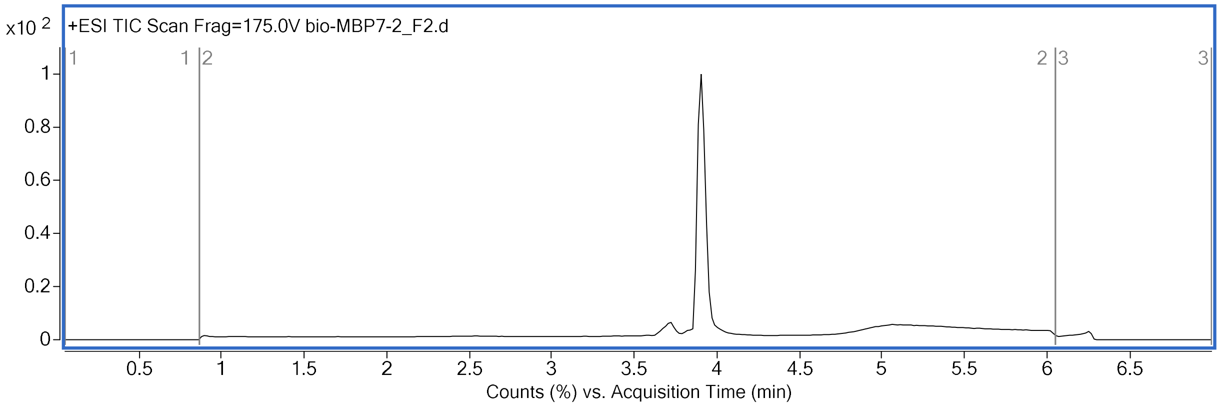

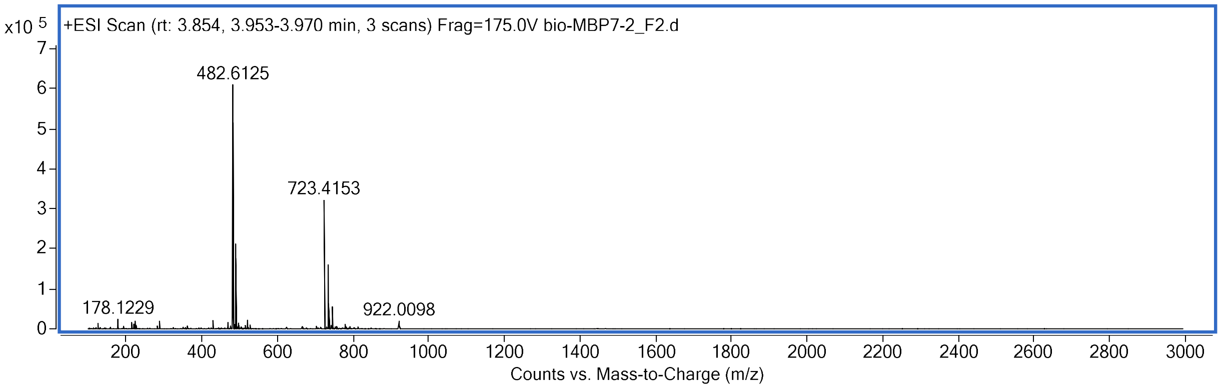

Biotin-hit 3

Sequence: Biotin-$(PEG)_4$-R(Napha)PVRPR (-0.98)

(Napha, 3-(2'-naphthyl)-L-alanine)

LC-MS Method: **Condition 2**

Observed: 1448.82 Da Calculated: 1448.81 Da

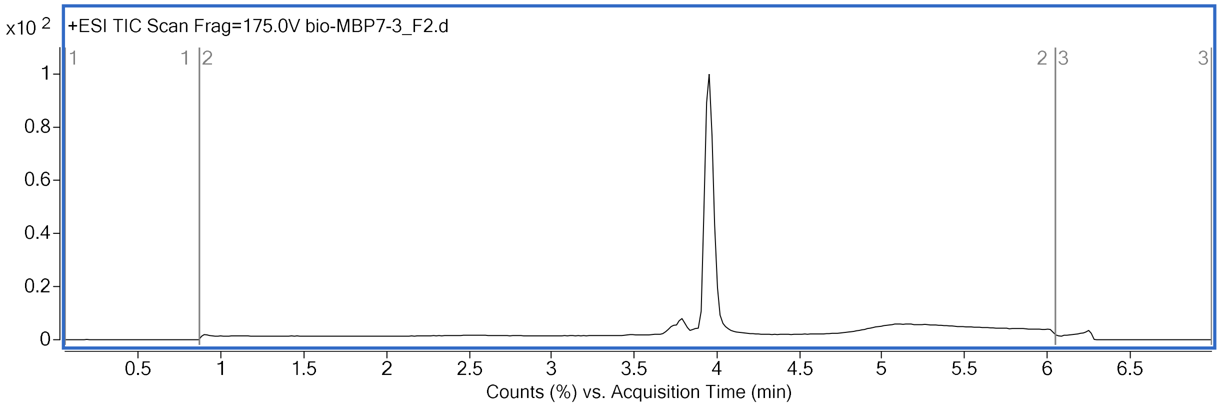

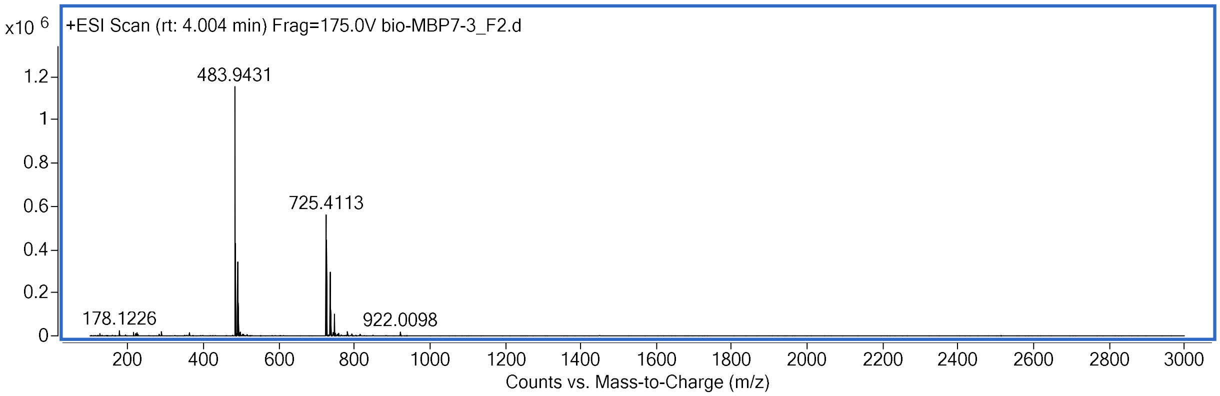

Biotin-hit 4

Sequence: Biotin-$(PEG)_4$-R(homof)(homoP)ARPR (-0.98)

(homof, L-homo-phenylalanine; homoP, L-homo-proline)

LC-MS Method: **Condition 2**

Observed: 1398.80 Da Calculated: 1398.79 Da

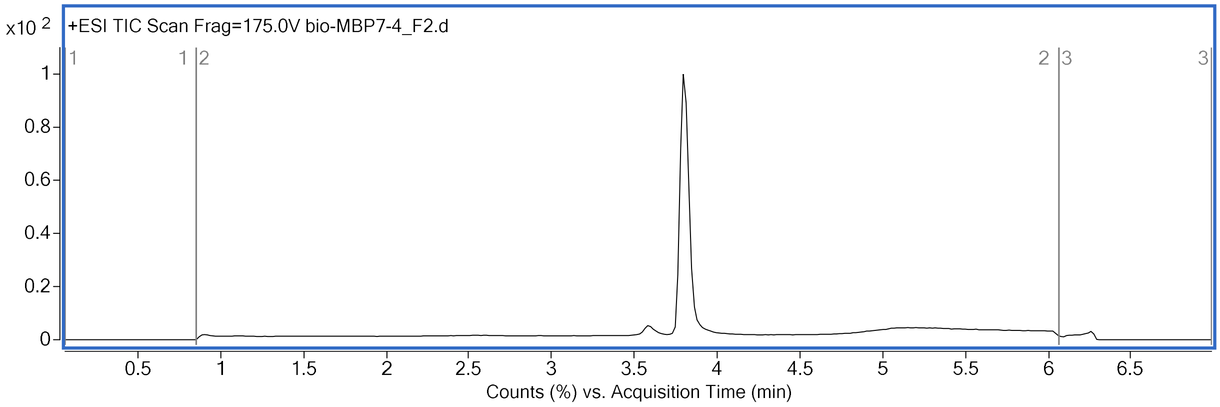

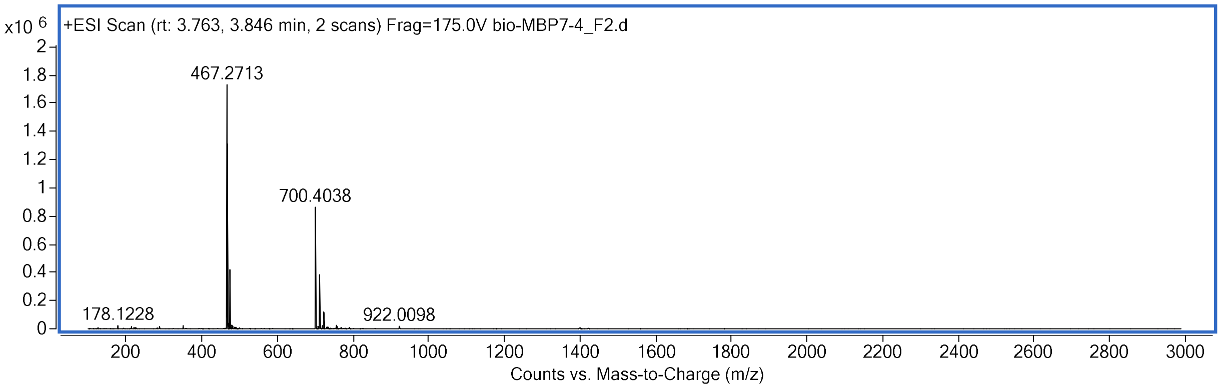

Biotin-hit 5

Sequence: Biotin-(PEG)4-R(Ff)P(Tle)RPR (-0.98)

(Ff, 4-fluoro-L-phenylalanine; Tle, L-α-tert-butylglycine)

LC-MS Method: **Condition 2**

Observed: 1430.81 Da Calculated: 1430.80 Da

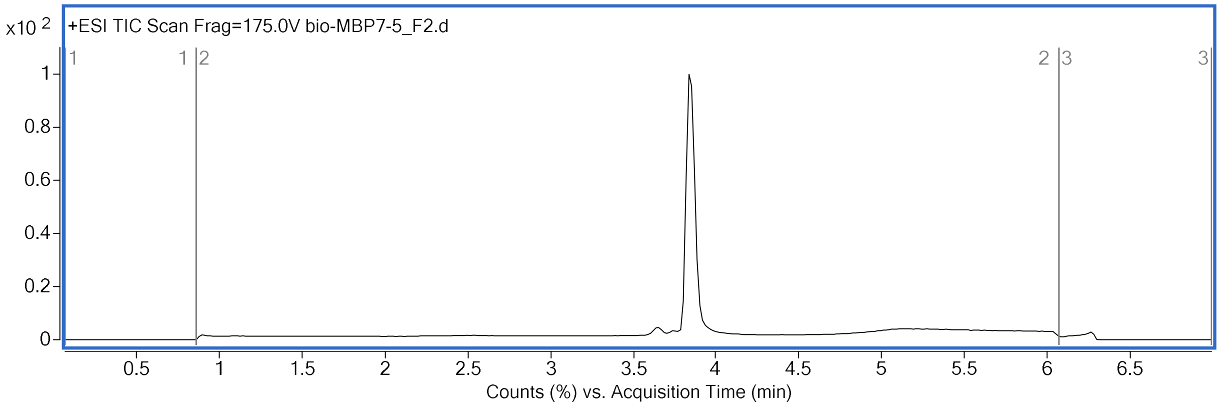

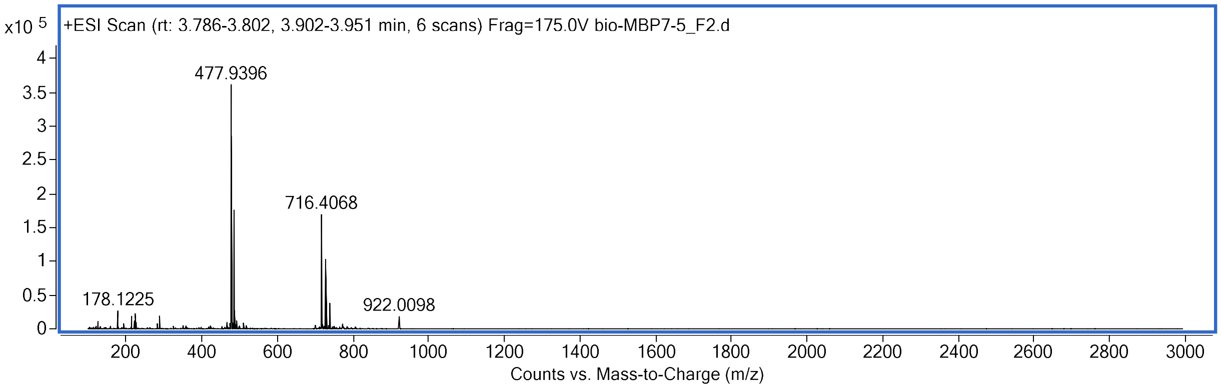

Biotin-hit 6

Sequence: Biotin-$(PEG)_4$-R(Cha)(homoP)VR(homoP)R (-0.98)

(Cha, β-cyclohexyl-L-alanine; homoP, L-homo-proline)

LC-MS Method: **Condition 2**

Observed: 1432.88 Da   Calculated: 1432.87 Da

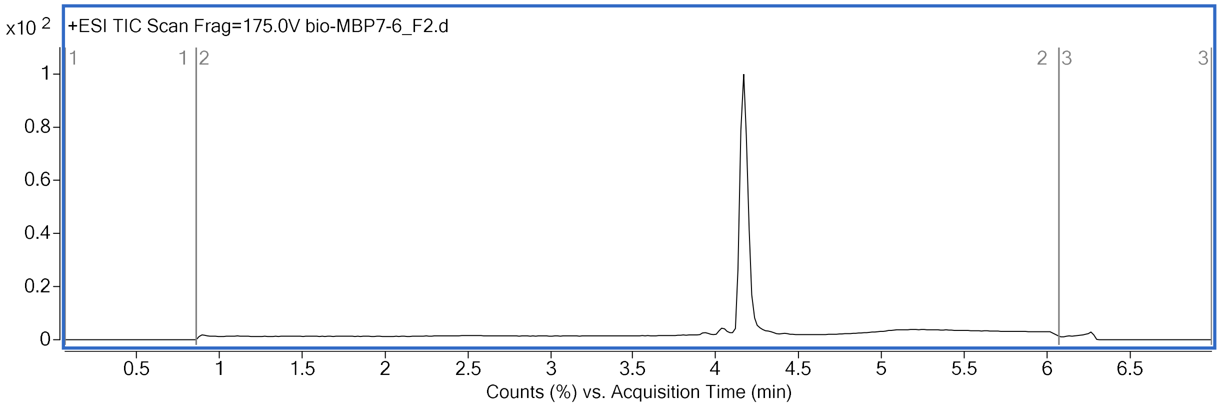

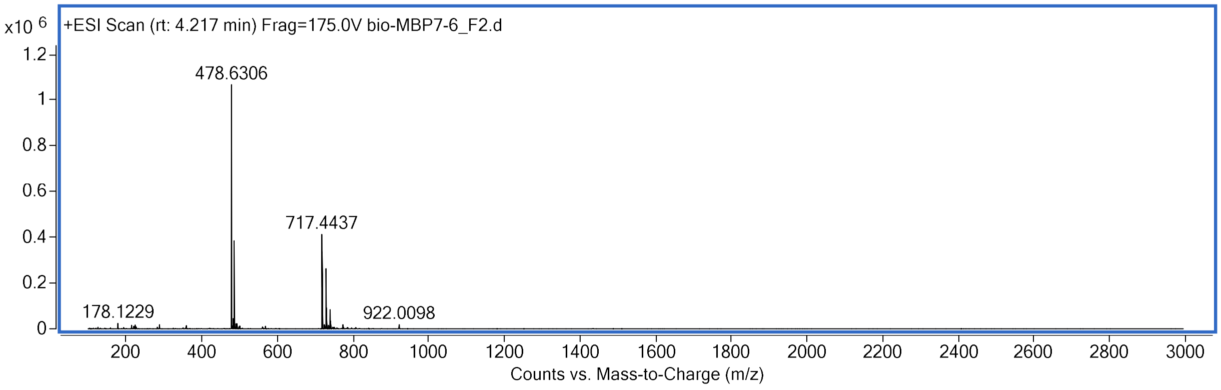

3.3.2 Biotin-hit 6-1 through 6-5

Biotin-hit 6-1

Sequence: Biotin-$(PEG)_4$-K(Cha)(homoP)V(SDMA)(homoP)R (-0.98)

(Cha, β-cyclohexyl-L-alanine; homoP, L-homo-proline; SDMA, symmetrical dimethylated arginine)

LC-MS Method: **Condition 2**

Observed: 1432.91 Da   Calculated: 1432.90 Da

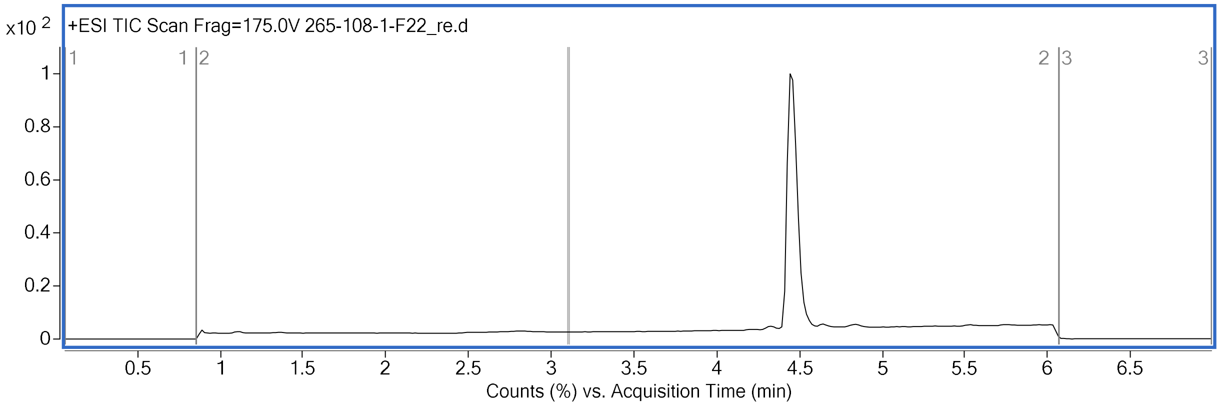

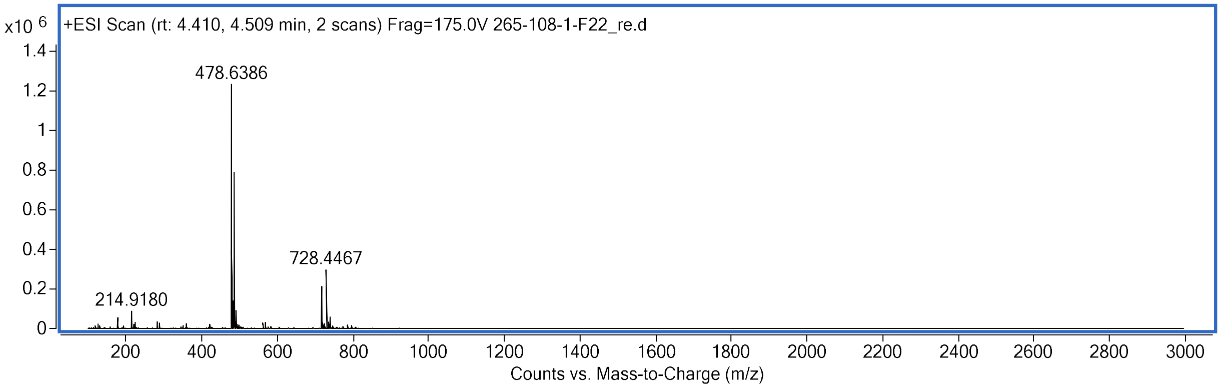

Biotin-hit 6-2

Sequence: Biotin-$(PEG)_4$-(Dab)(Cha)(homoP)VR(homoP)(Amf) (-0.98)

(Dab, L-2,4-Diaminobutyric acid; Cha, β-cyclohexyl-L-alanine; homoP, L-homo-proline; Amf, 4-(amino-methyl)-L-phenylalanine)

LC-MS Method: **Condition 2**

Observed: 1396.84 Da Calculated: 1396.83 Da

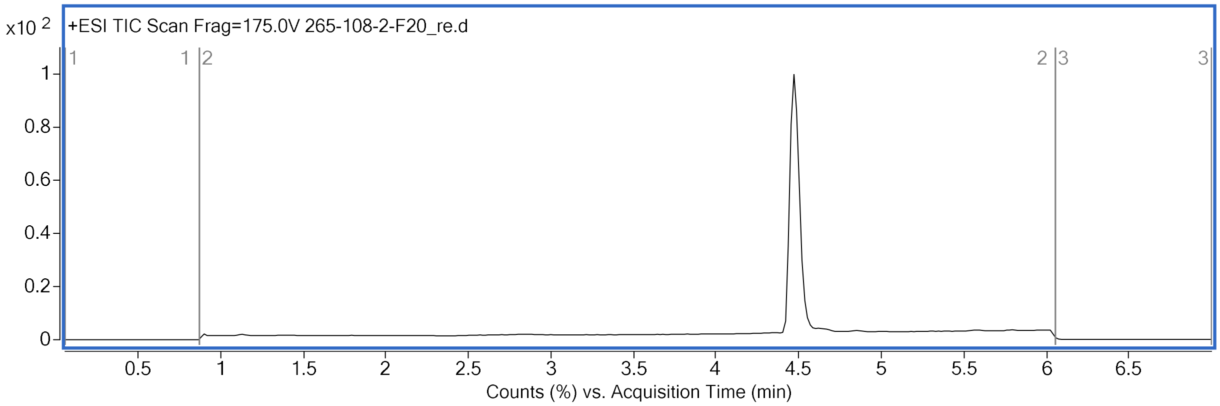

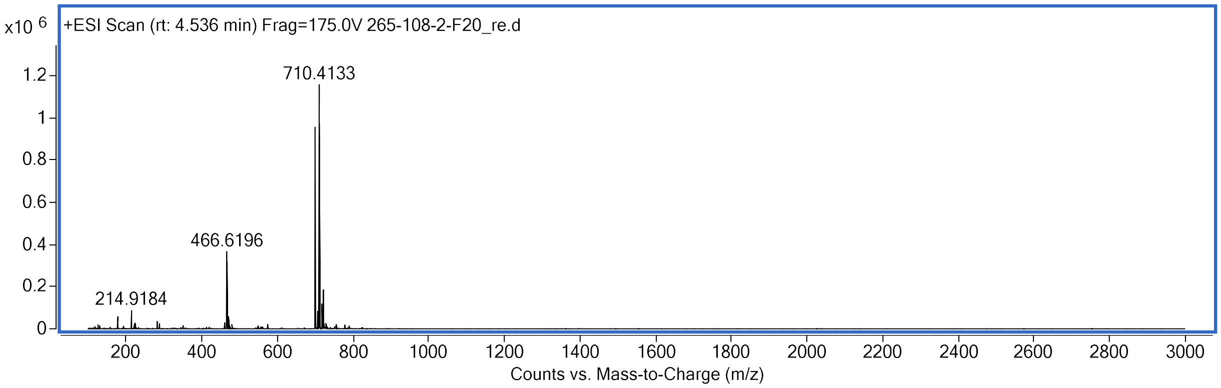

Biotin-hit 6-3

Sequence: Biotin-$(PEG)_4$-(Dab)(Cha)(homoP)V(hAr)(homoP)R (-0.98)

(Dab, L-2,4-Diaminobutyric acid; Cha, β-cyclohexyl-L-alanine; homoP, L-homo-proline; hAr, L-homoarginine)

LC-MS Method: **Condition 2**

Observed: 1390.86 Da   Calculated: 1390.85 Da

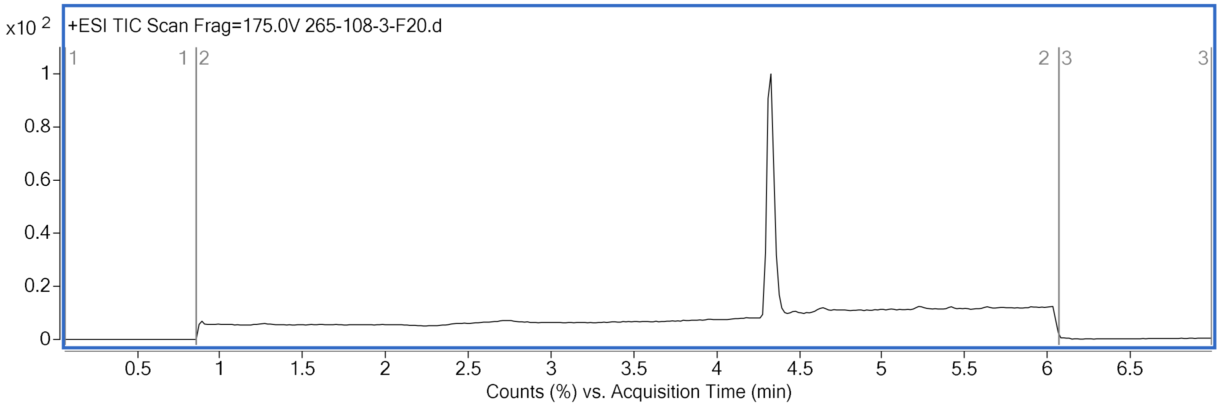

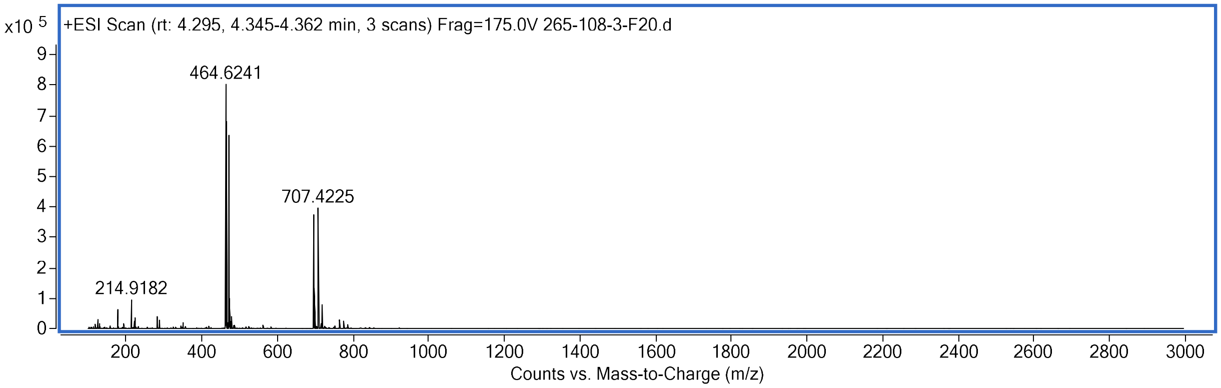

Biotin-hit 6-4

Sequence: Biotin-(PEG)$_4$-K(Cha)(homoP)V(mLys)(homoP)R (-0.98)

(Cha, β-cyclohexyl-L-alanine; homoP, L-homo-proline; mLys, L-Lys(Me))

LC-MS Method: **Condition 2**

Observed: 1390.89 Da Calculated: 1390.87 Da

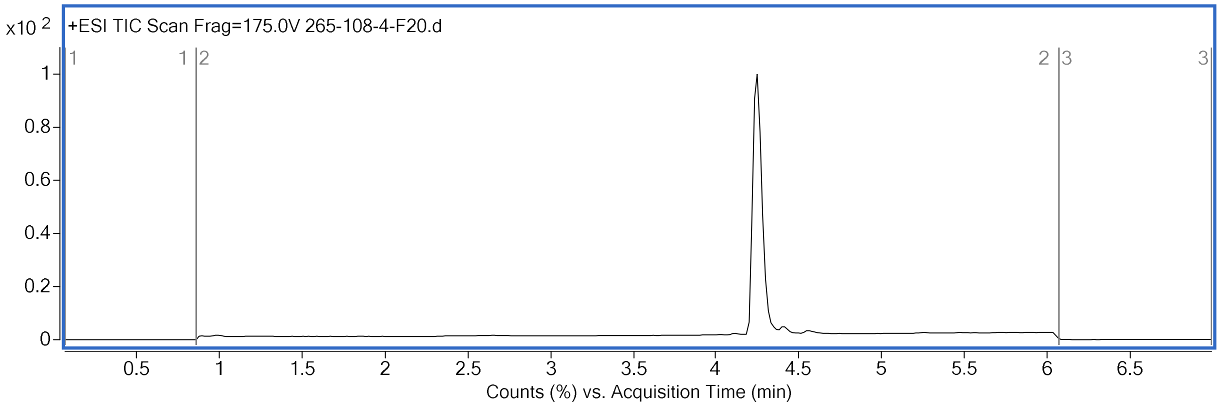

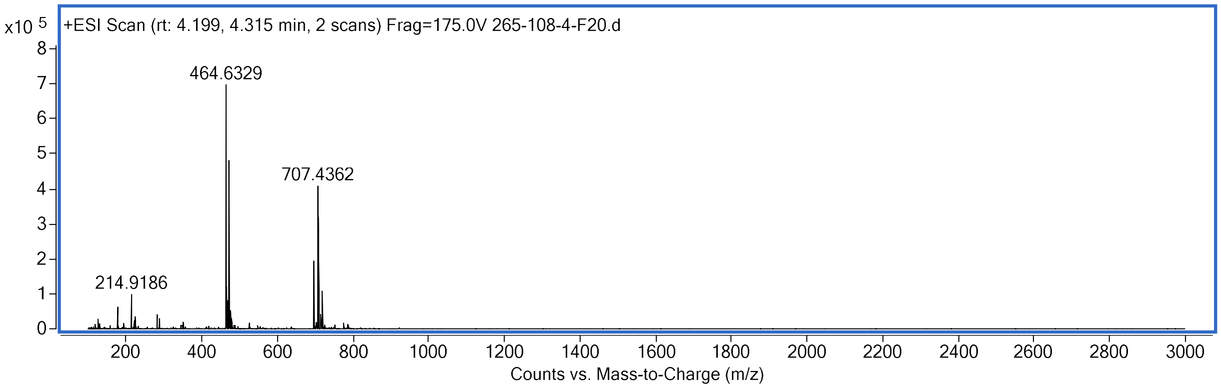

Biotin-hit 6-5

Sequence: Biotin-(PEG)$_4$-H(Cha)(homoP)V(mLys)(homoP)R (-0.98)

(Cha, β-cyclohexyl-L-alanine; homoP, L-homo-proline; mLys, L-Lys(Me))

LC-MS Method: **Condition 2**

Observed: 1399.85 Da Calculated: 1399.84 Da

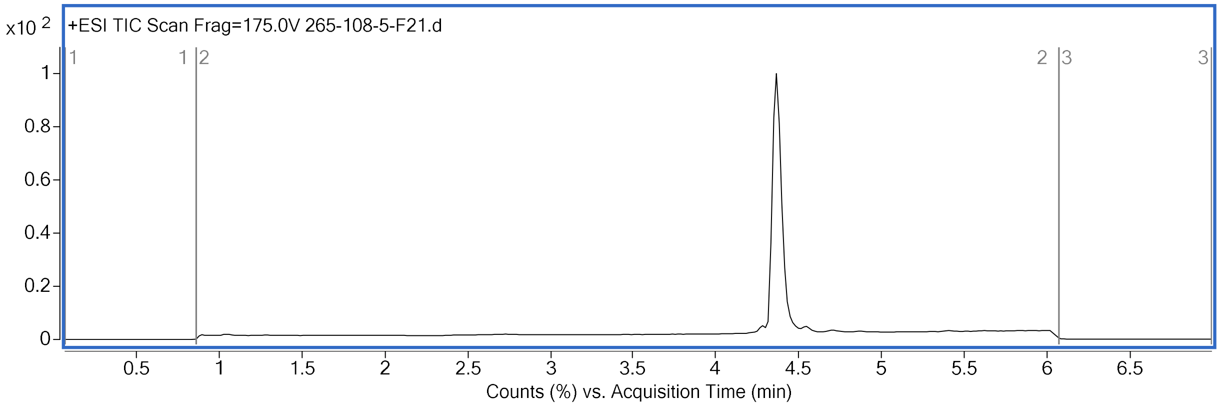

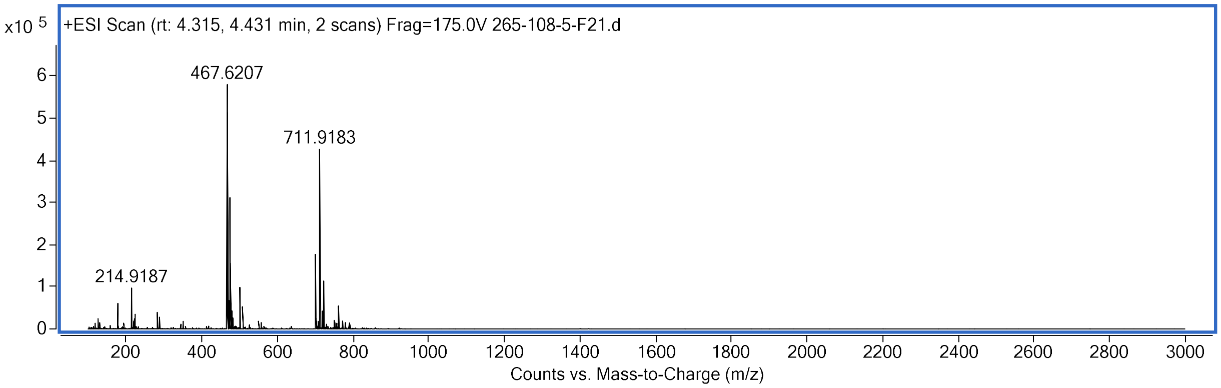

4. Library preparation and QC

4.1 Three randomized libraries

The synthesis, description, and characterization of the 2×10^6, 2×10^7, and 2×10^8-member libraries used in this study are reported in our recent article[2], of which the supporting information can be found at the link below:

https://static-content.springer.com/esm/art%3A10.1038%2Fs41467-020-16920-3/MediaObjects/41467_2020_16920_MOESM1_ESM.pdf

4.2 Focused libraries 1 and 2

Both focused libraries 1 and 2 were synthesized following the procedures specified in the Method Section. After completing the library synthesis, 5.0 mg of peptide-loaded resin were cleaved, and the theoretical amount of peptides were calculated based on the resin loading and averaged peptide molecular weight. Subsequently, solid-phase extraction was performed on the cleaved peptides followed by nLC-MS/MS sequencing analysis. The PEAKS software suite was used to decode the peptide sequences, and all monomers at each position were found approximately evenly distributed in the library peptides. Note: due to the large size of the dataset, the original library quality control sequencing data was not shown, but are available upon request.

5. BLI-assisted library screen and affinity enrichment

Bio-layer interferometry (BLI) is an optical analytical technique developed for binding characterizations of biomolecules. BLI is designed for specific binding measurements with high sensitivity, and multiple samples (8 or 16, depending on the format of plates being used) can be analyzed simultaneously. In our study, the BLI technique is repurposed for peptide library selection and detailed experimental examples can be found below.

5.1 Procedures of BLI-assisted library screen

A typical BLI-assisted library screen procedure includes five steps, and the screenshots of each step were provided below. After completing the whole procedure, eluted fractions were retrieved manually and processed to peptide sequencing.

Step 1: Define the plate. The example below is a side-by-side library screening against 12ca5 (row A, B, and C) and menin proteins (row D, E, and F). Column 1, 3 and 5 contain 1 × kinetic buffer, column 2 contains proteins (A2-C2: 12ca5, and D2-F2: menin), column 4 contains the peptide library (at concentrations 100 nM, 10 nM, and 1 nM per peptide), and column 6 contains the elution buffer (6.0 M Guanidine·HCl, pH 6.8).

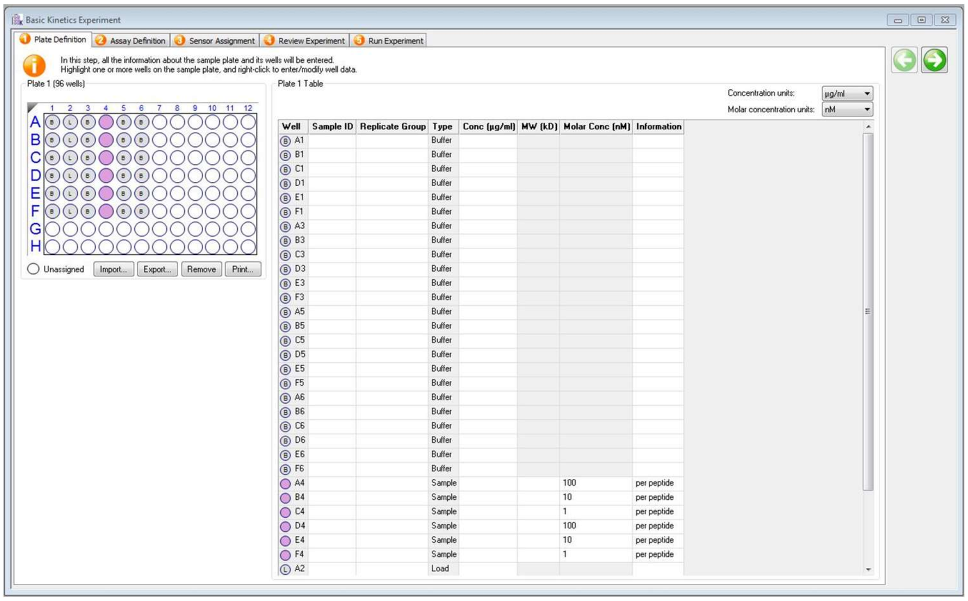

Step 2: Define the assay steps. A typical assay run includes: baseline 1 (60 s), protein loading (300-600 s), baseline 2 (120 s), library incubation (30 min), baseline 3 (20 s) and elution (300 s). Total run time: ~45 min.

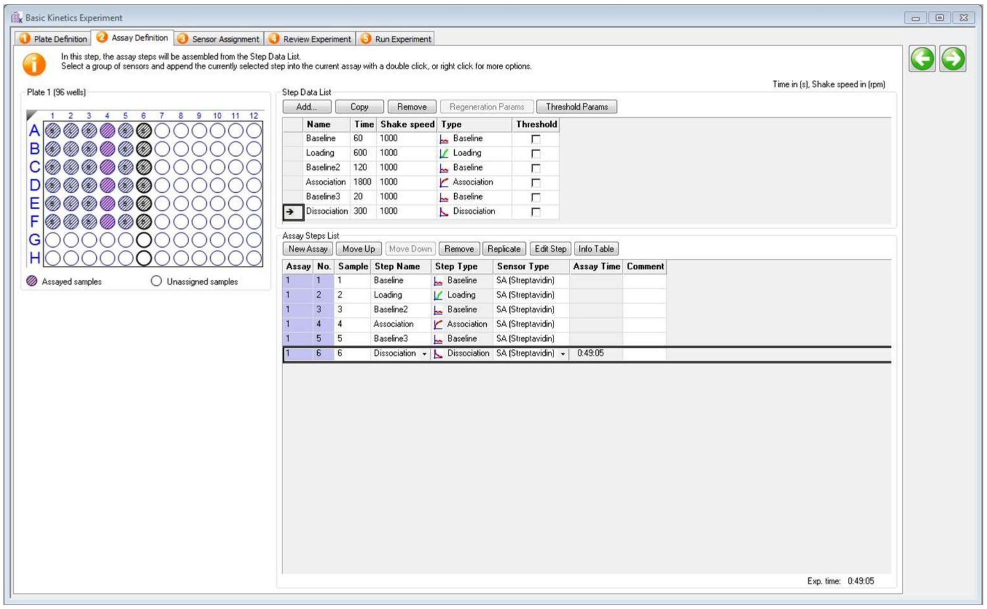

Step 3: Assign the locations of streptavidin-coated biosensors.

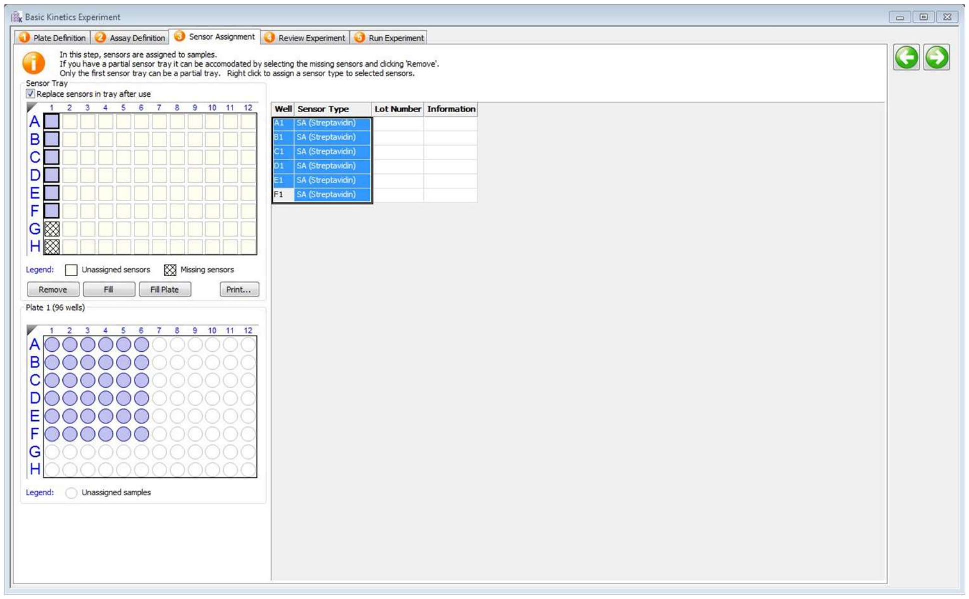

Step 4: Review the experimental steps.

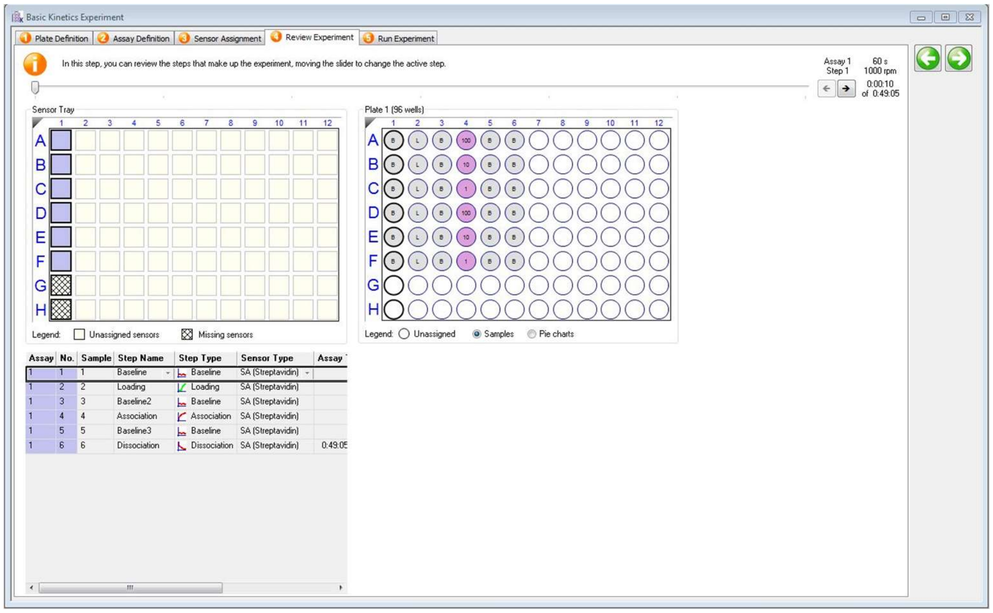

Step 5: Specify saving file location and check run settings. Then hit 'Go' to start.

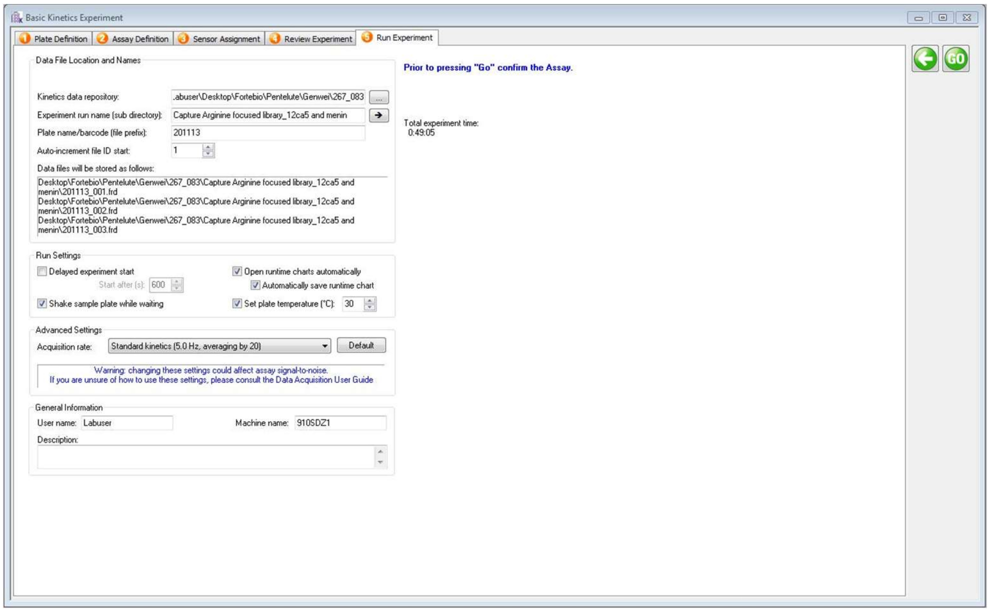

5.2 Indirect capture of HA tag using BLI AS-MS

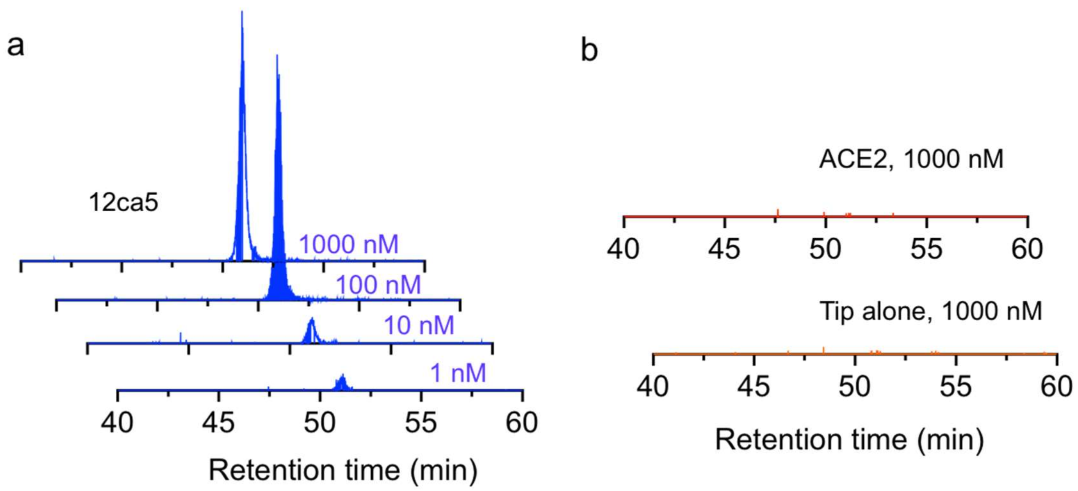

Figure S3. The extracted-ion chromatogram (m/z = 615.29-615.30) of the HA epitope (sequence: YPYDVPDYAK) after mass spectrometry analysis of screens against 12ca5 protein (**panel a**, at peptide concentrations 1.0 µM, 100 nM, 10 nM and 1.0 nM). Both an unrelated protein (human ACE2) and the streptavidin-coated tip alone were used as controls (**panel b)** at the highest peptide concentration of 1.0 µM. HA epitope was enriched in 12ca5 at all tested concentrations. After nLC-MS/MS analysis, decreased ion intensities were observed as the input peptide concentration decreased, but no enrichment was found in either control. Of note, at the concentration of 1.0 nM per peptide, the PEAKS software confidently assigns the HA epitope sequence correctly after nLC-MS/MS analysis.

5.3 Recovery of tMBM1.0 sequence using BLI AS-MS

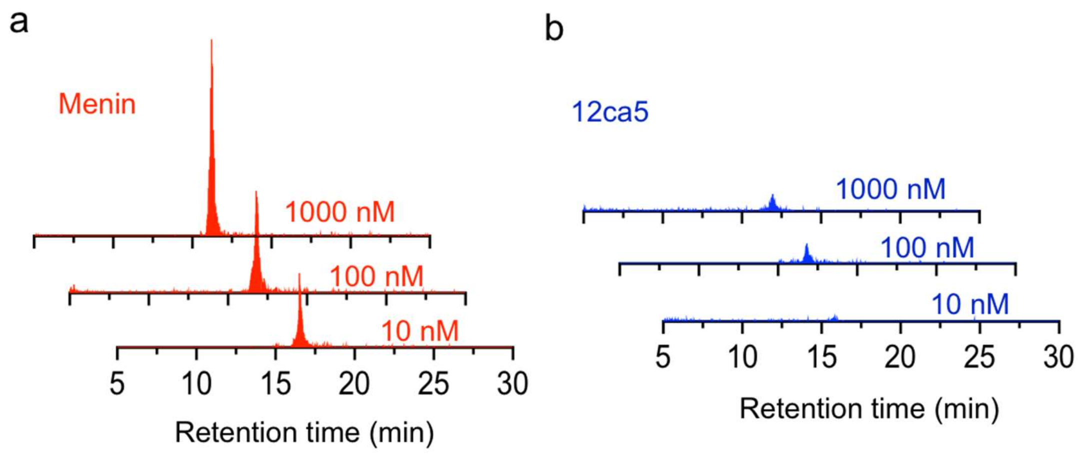

Figure S4. The extracted-ion chromatogram (m/z = 310.93-310.94) of the tMBM1.0 peptide (sequence: RWRFPARPR) after mass spectrometry analysis of the library screens against protein menin (**panel a**) and the control protein 12ca5 (**panel b).** Peptide concentrations used are 1000 nM, 100 nM and 10 nM. The tMBM1.0 peptide was enriched towards menin at all three tested concentrations with decreased ion intensities when the input amount of peptides was decreased, and negligible enrichment was found in the control. Of note, at condition 1000 nM per peptide in the control run, software PEAKS starts assigning ions to the peptide sequence of interest, but with a low averaged local residue confidence (ALC score was ~40%), indicating that nonspecific binding starts emerging at high concentrations. However, no such phenomenon was observed at or below 100 nM per peptide. To minimize the nonspecific binding, within all the subsequent library screens, per peptide concentration was kept at concentrations not exceeding 100 nM per peptide.

5.4 Extracted ions of enriched peptide from library 1

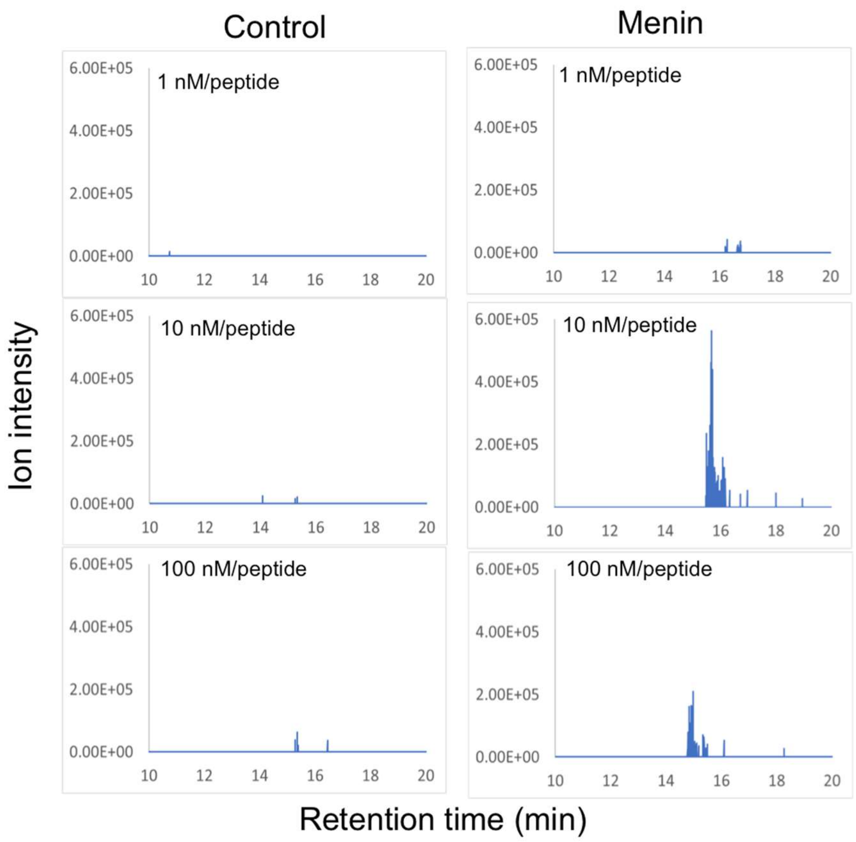

Figure S5. The extracted-ion chromatogram (m/z = 311.54-311.55) of **hit 1** (sequence: R(Cba)(homoP)VR(homoP)R) after mass spectrometry analysis of the BLI screens against menin (**right panels**) and the 12ca5 control protein (**left panels**).

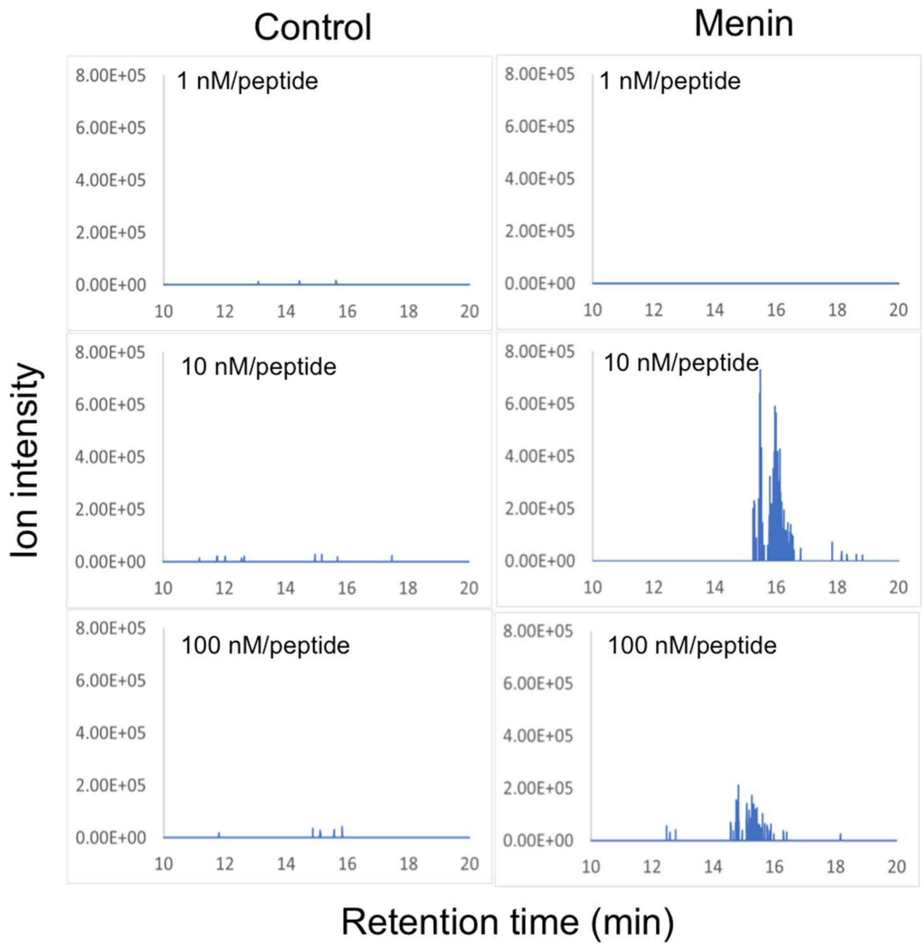

Figure S6. The extracted-ion chromatogram (m/z = 324.87-324.88) of **hit 2** (sequence: R(homof)P(Tle)R(4FP)R) after mass spectrometry analysis of BLI screens against menin (**right panels**) and the 12ca5 control protein (**left panels**).

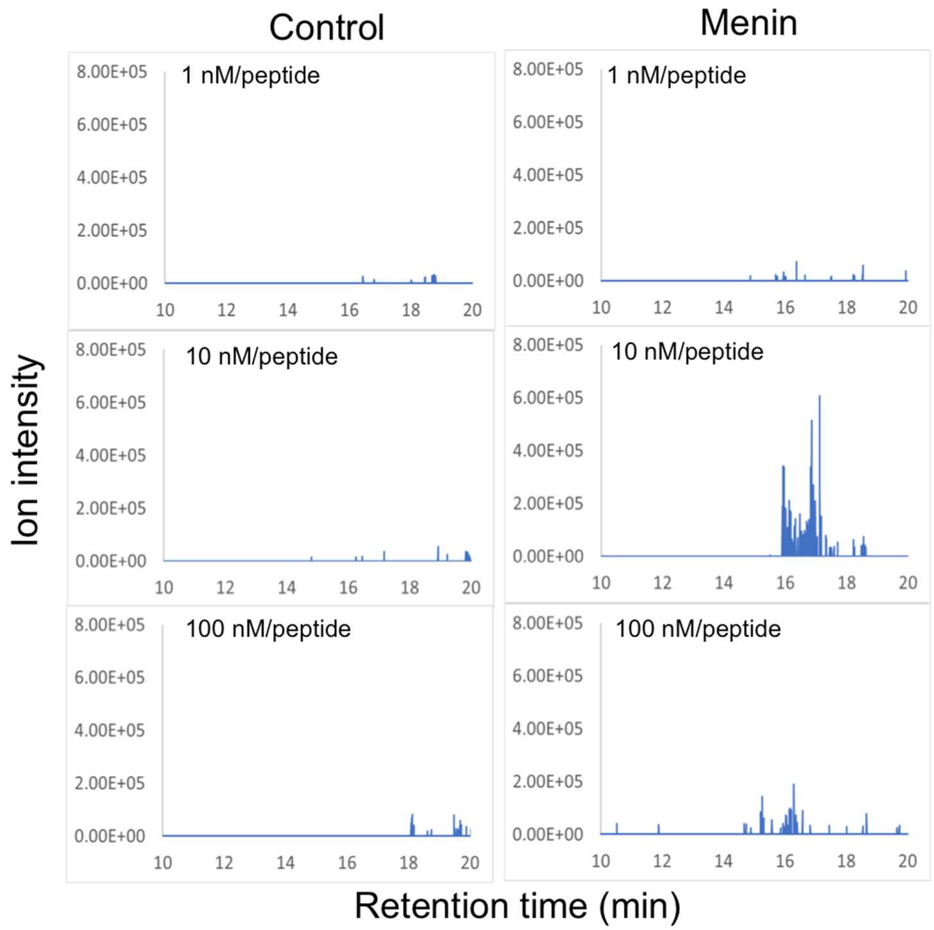

Figure S7. The extracted-ion chromatogram (m/z = 326.20-326.21) of **hit 3** (sequence: R(Napha)PVRPR) after mass spectrometry analysis of BLI screens against menin (**right panels**) and the 12ca5 control protein (**left panels**).

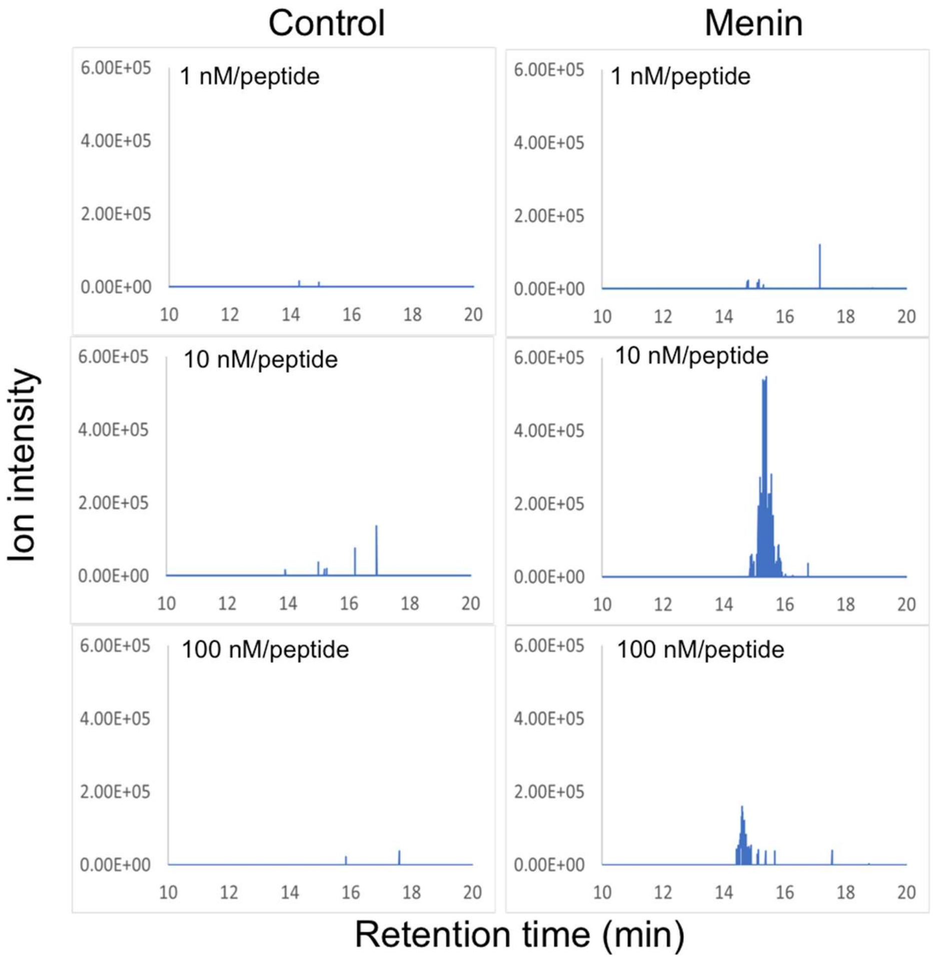

Figure S8. The extracted-ion chromatogram (m/z = 309.53-309.54) of **hit 4** (sequence: R(homof)(homoP)ARPR) after mass spectrometry analysis of BLI screens against menin (**right panels**) and the 12ca5 control protein (**left panels**).

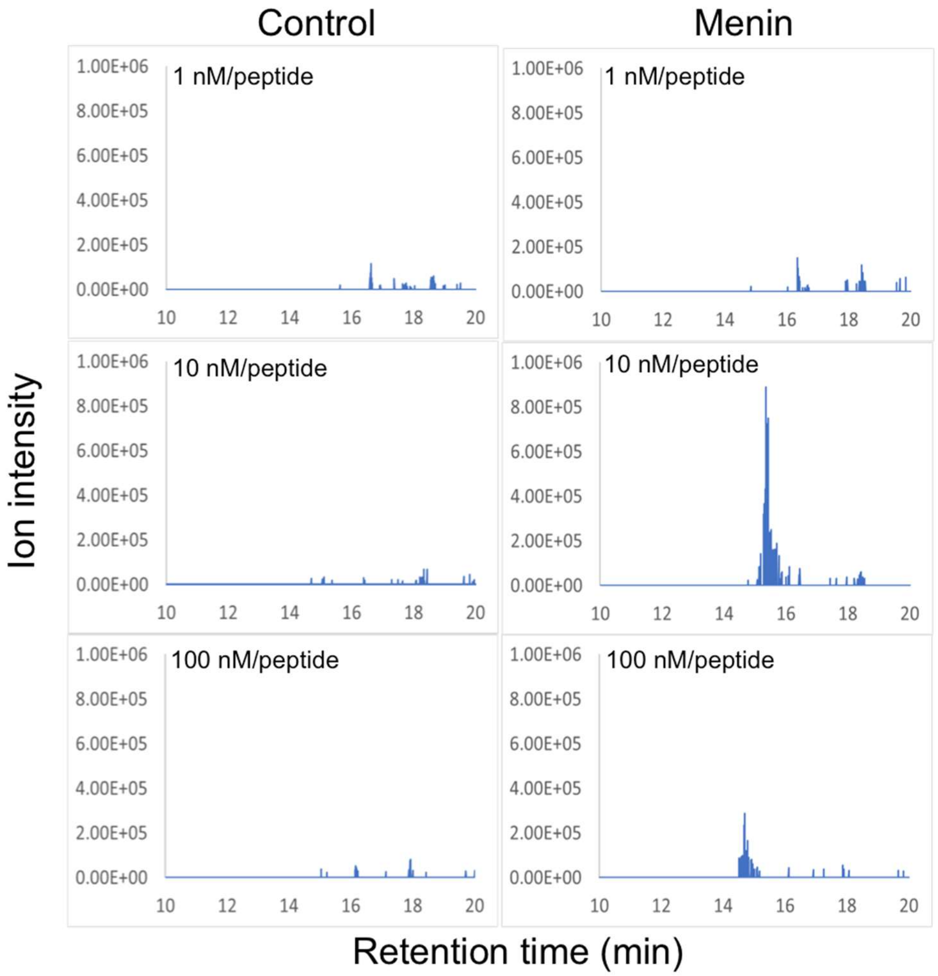

Figure S9. The extracted-ion chromatogram (m/z = 320.20-320.21) of **hit 5** (sequence: R(4Ff)P(Tle)RPR) after mass spectrometry analysis of BLI screens against menin (**right panels**) and the 12ca5 control protein (**left panels**).

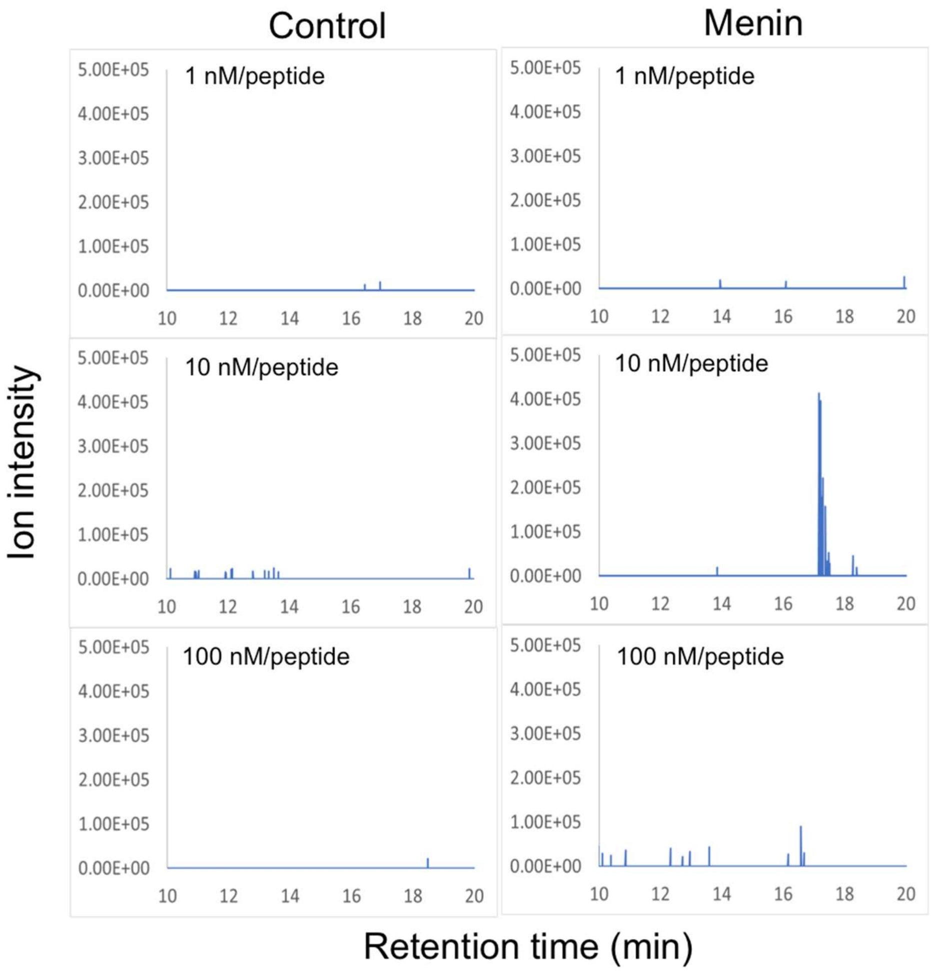

Figure S10. The extracted-ion chromatogram (m/z = 320.89-320.90) of **hit 6** (sequence: R(Cha)(homoP)VR(homoP)R) after MS analysis of BLI screens against menin (**right panels**) and the 12ca5 control protein (**left panels**).

5.5 Extracted ions of enriched peptide from library 2

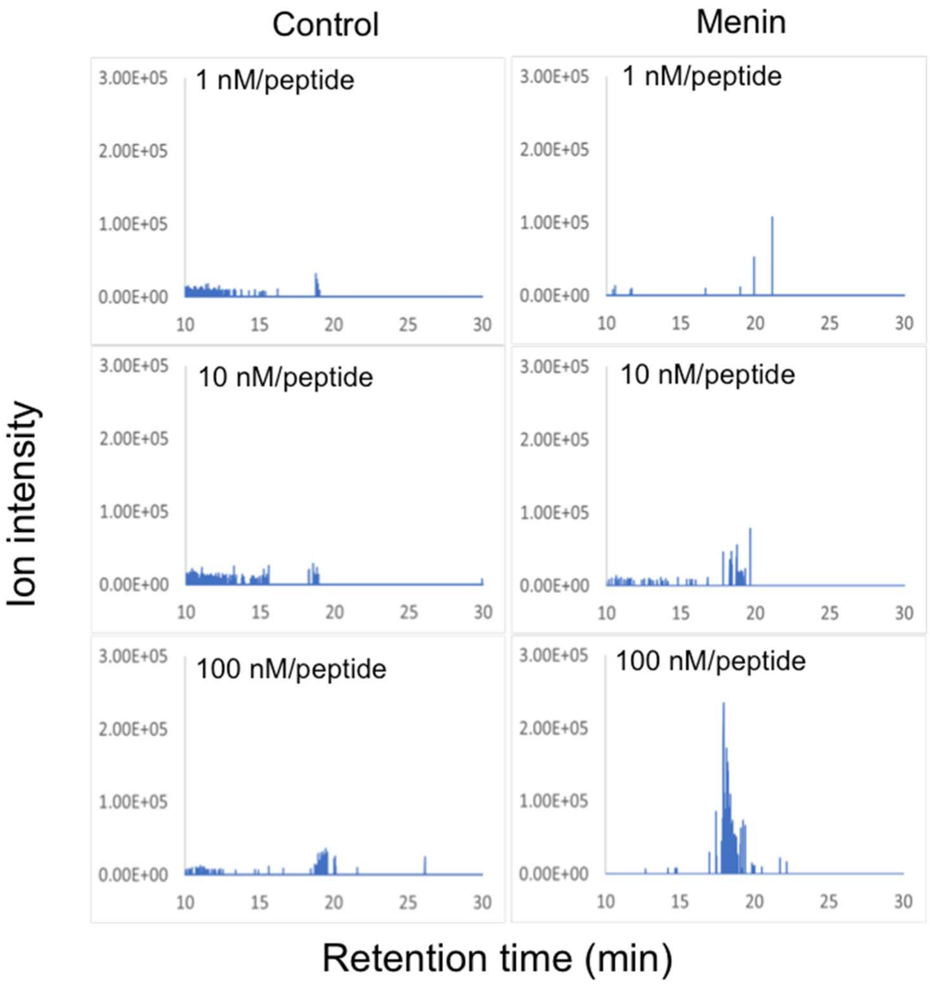

Figure S11. The extracted-ion chromatogram (m/z = 320.89-320.90) of **hit 6-1** (sequence: K(Cha)(homoP)V(SDMA)(homoP)R) after mass spectrometry analysis of BLI screens against menin (**right panels**) and the 12ca5 control protein (**left panels**).

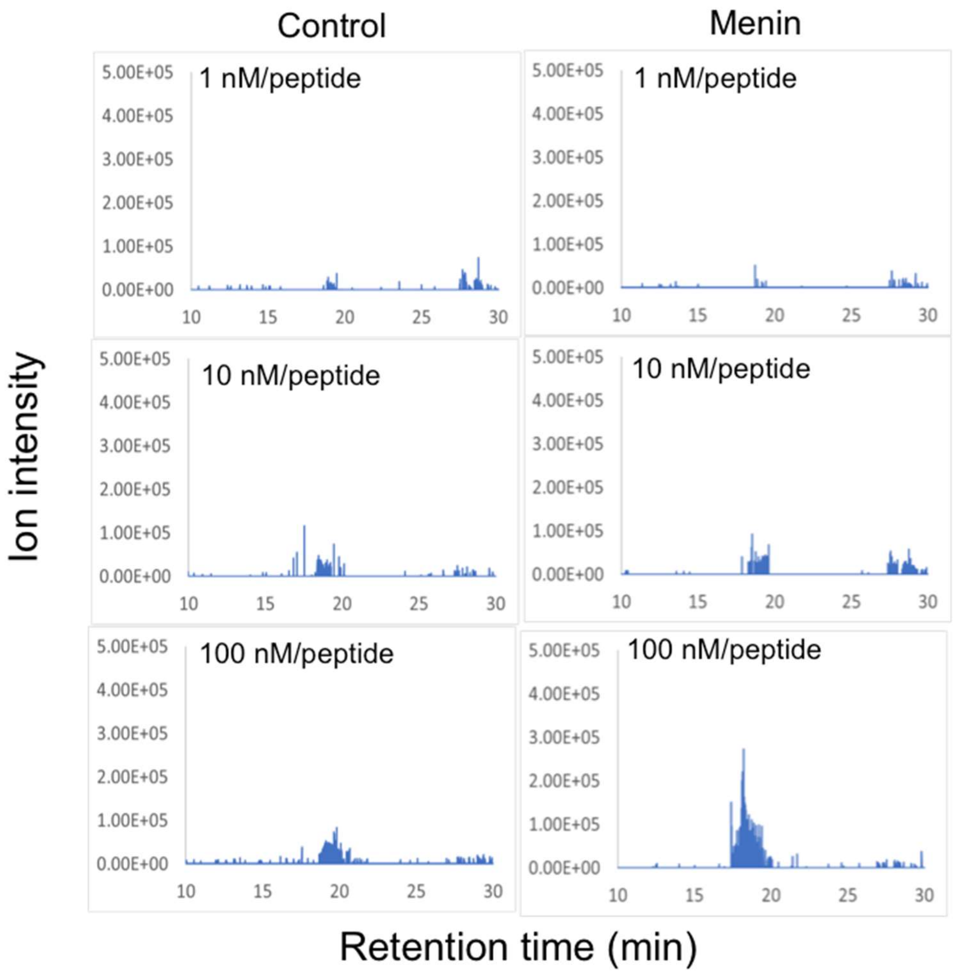

Figure S12. Dab)(Cha)(homoP)VR(homoP)(Amf)) after mass spectrometry analysis of BLI screens against menin (**right panels**) and the 12ca5 control protein (**left panels**).

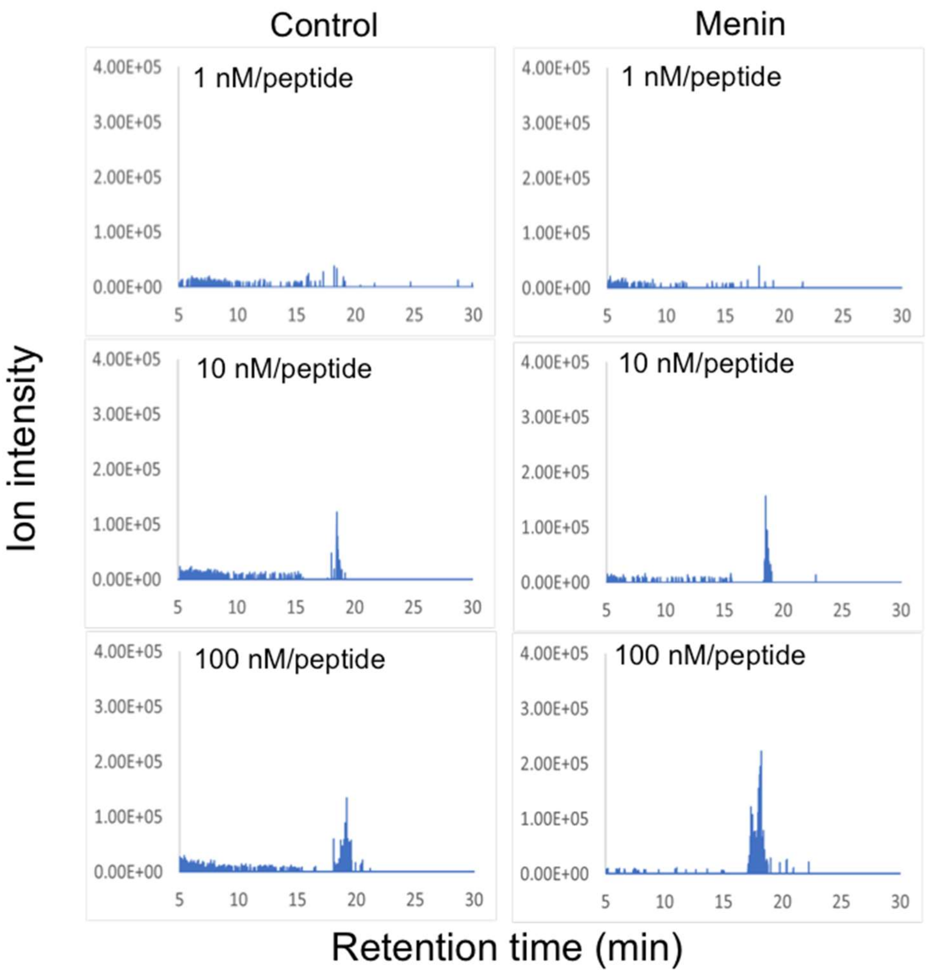

Figure S13. The extracted-ion chromatogram (m/z = 306.88-306.89) of **hit 6-3** (sequence: (Dab)(Cha)(homoP)V(hAr)(homoP)R) after mass spectrometry analysis of BLI screens against menin (**right panels**) and the 12ca5 control protein (**left panels**).

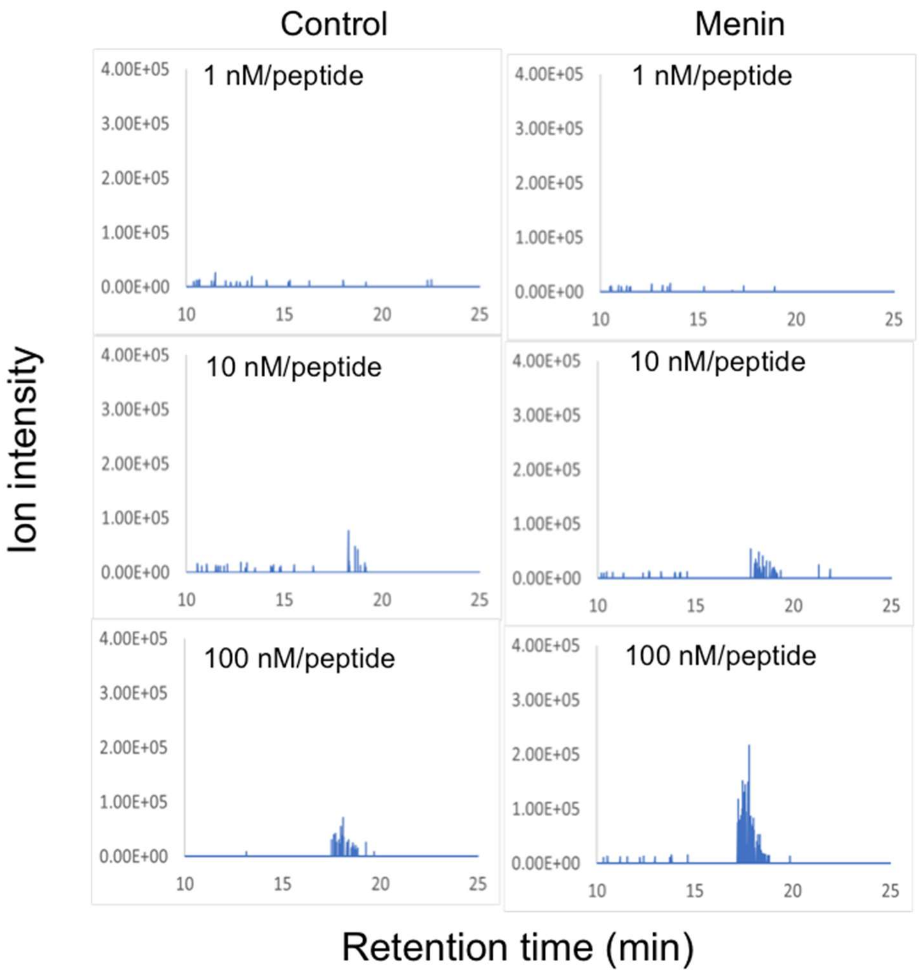

Figure S14. The extracted-ion chromatogram (m/z = 306.89-306.90) of **hit 6-4** (sequence: K(Cha)(homoP)V(mLys)(homoP)R) after mass spectrometry analysis of BLI screens against menin (**right panels**) and the ACE2 control protein (**left panels**).

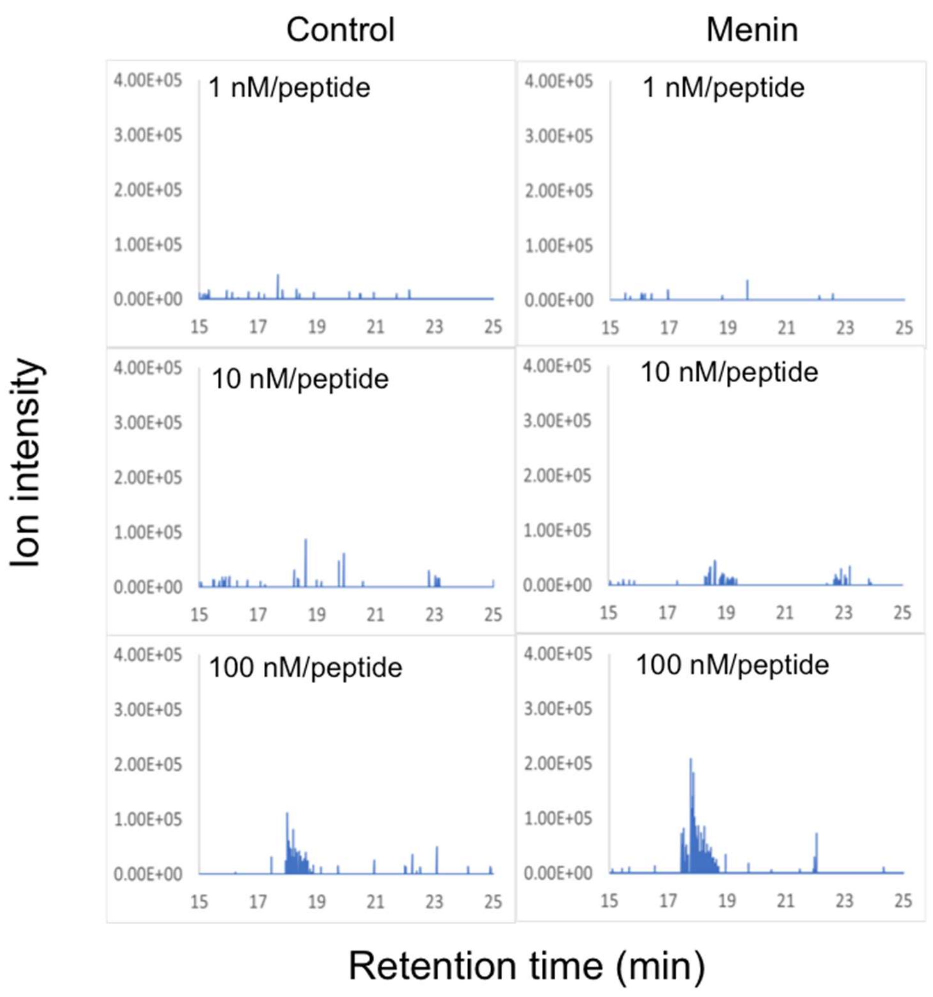

Figure S15. The extracted-ion chromatogram (m/z = 309.87-309.89) of **hit 6-5** (sequence: H(Cha)(homoP)V(mLys)(homoP)R) after mass spectrometry analysis of BLI screens against menin (**right panels**) and the ACE2 control protein (**left panels**).

6. Binding affinity measurement of library screen hits using BLI

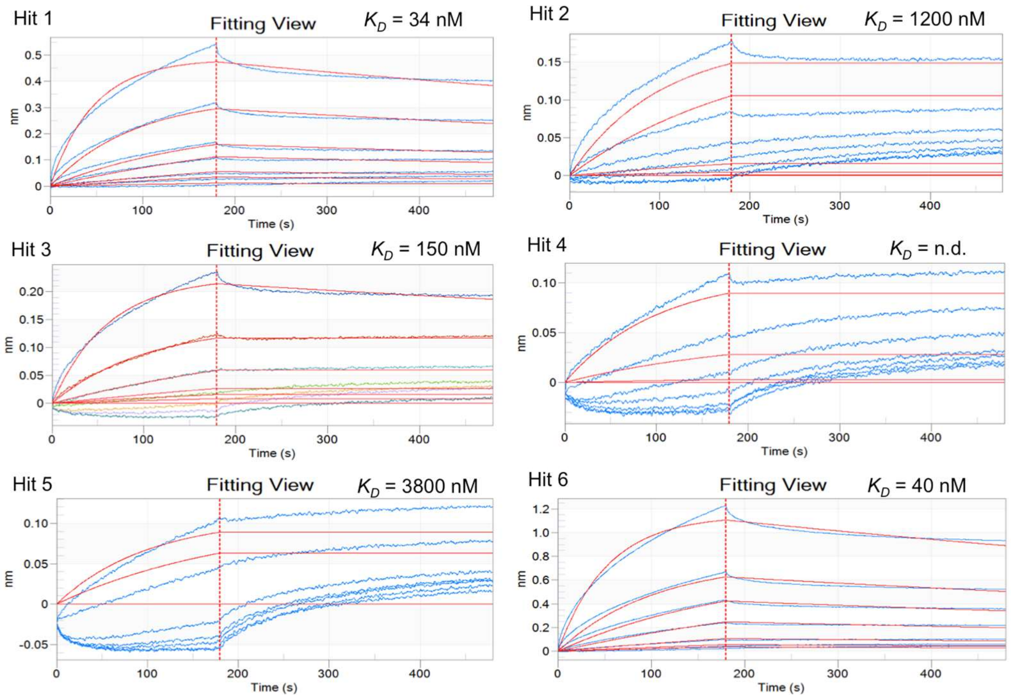

Figure S16. The binding curve-fitting view of **hit 1** to **hit 6** from Library 1 BLI screen against menin. The apparent binding affinity was reported from the 1:1 mode kinetic fitting results (K_D is calculated by dividing the off-rate by on-rate) for **hit 1** and **hit 6**. The concentrations of recombinant menin protein used are 1000, 500, 250, 125, 62.5, 31.3, and 15.6 nM. However, due to the large fitting error for **hits 2, 3, 4** and **5**, binding affinity is reported based on the steady-state calculations, as an approximation. Of note, we observed that the calculated binding affinity of **hit 4** was an exceedingly large value (>6.6E8 M), and as such we consider **hit 4** as non-binding.

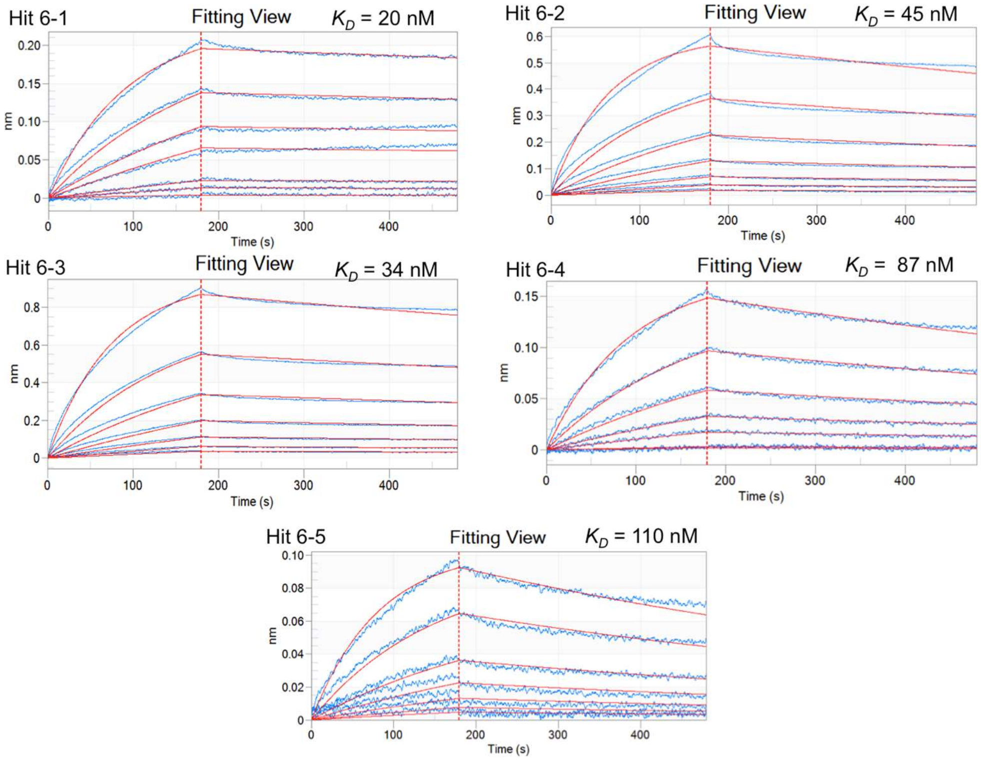

Figure S17. The binding curve-fitting view of **hit 6-1** to **hit 6-5** from Library 2 BLI screens against menin. The apparent binding affinity was reported from the 1:1 mode kinetic fitting results (K_D is calculated by dividing the off-rate by on-rate). The concentrations of recombinant menin protein used are 1000, 500, 250, 125, 62.5, 31.3, and 15.6 nM.

7. References

1. Simon, M. D.; Heider, P. L.; Adamo, A.; Vinogradov, A. A.; Mong, S. K.; Li, X. Y.; Berger, T.; Policarpo, R. L.; Zhang, C.; Zou, Y. K.; Liao, X. L.; Spokoyny, A. M.; Jensen, K. F.; Pentelute, B. L., Rapid Flow- Based Peptide Synthesis. *Chembiochem* **2014,** *15* (5), 713-720.

2. Quartararo, A. J.; Gates, Z. P.; Somsen, B. A.; Hartrampf, N.; Ye, X. Y.; Shimada, A.; Kajihara, Y.; Ottmann, C.; Pentelute, B. L., Ultra-large chemical libraries for the discovery of high-affinity peptide binders. *Nat. Commun.* **2020,** *11* (1).
